# Genomic and metatranscriptomic analyses of carbon remineralization in an Antarctic polynya

**DOI:** 10.1186/s40168-019-0643-4

**Published:** 2019-02-20

**Authors:** So-Jeong Kim, Jong-Geol Kim, Sang-Hoon Lee, Soo-Je Park, Joo-Han Gwak, Man-Young Jung, Won-Hyung Chung, Eun-Jin Yang, Jisoo Park, Jinyoung Jung, Yoonsoo Hahn, Jang-Cheon Cho, Eugene L. Madsen, Francisco Rodriguez-Valera, Jung-Ho Hyun, Sung-Keun Rhee

**Affiliations:** 1 Geologic Environment Research Division, Korea Institute of Geoscience and Mineral Resources, Daejeon, 34132 Republic of Korea; 2 Department of Microbiology, Chungbuk National University, Cheongju, 28644 Republic of Korea; 3 Division of Polar Ocean Environment, Korea Polar Research Institute, Incheon, 21990 Republic of Korea; 4 Department of Biology, Jeju National University, Jeju, 63243 Republic of Korea; 5 Department of Microbial Ecology, University of Vienna, 1090 Vienna, Austria; 6 Research Group of Gut Microbiome, Korea Food Research Institute, Sungnam, 13539 Republic of Korea; 7 Department of Life Science, Chung-Ang University, Seoul, 06974 Republic of Korea; 8 Department of Biological Sciences, Inha University, Incheon, 22212 Republic of Korea; 9 Department of Microbiology, Cornell University, Ithaca, NY 14853-8101 USA; 10 Evolutionary Genomics Group, División de Microbiología, Universidad Miguel Hernández, Apartado 18, San Juan de Alicante, 03550 Alicante, Spain; 11 Department of Marine Science and Convergence Engineering, Hanyang University ERICA Campus, Ansan, 15588 Republic of Korea

**Keywords:** Carbon remineralization, Genomics, Metatranscriptomics, Polynya

## Abstract

**Background:**

Polynyas in the Southern Ocean are regions of intense primary production, mainly by *Phaeocystis antarctica*. Carbon fixed by phytoplankton in the water column is transferred to higher trophic levels, and finally, to the deep ocean. However, in the Amundsen Sea, most of this organic carbon does not reach the sediment but is degraded in the water column due to high bacterial heterotrophic activity.

**Results:**

We reconstructed 12 key bacterial genomes from different phases of bloom and analyzed the expression of genes involved in organic carbon remineralization. A high correlation of gene expression between the peak and decline phases was observed in an individual genome bin-based pairwise comparison of gene expression. *Polaribacter* belonging to *Bacteroidetes* was found to be dominant in the peak phase, and its transcriptional activity was high (48.9% of the total mRNA reads). Two dominant *Polaribacter* bins had the potential to utilize major polymers in *P. antarctica*, chrysolaminarin and xylan, with a distinct set of glycosyl hydrolases. In the decline phase, *Gammaproteobacteria* (Ant4D3, SUP05, and SAR92), with the potential to utilize low molecular weight-dissolved organic matter (LMW-DOM) including compatible solutes, was increased. The versatility of *Gammaproteobacteria* may contribute to their abundance in organic carbon-rich polynya waters, while the SAR11 clade was found to be predominant in the sea ice-covered oligotrophic ocean. SAR92 clade showed transcriptional activity for utilization of both polysaccharides and LMW-DOM; this may account for their abundance both in the peak and decline phases. Ant4D3 clade was dominant in all phases of the polynya bloom, implicating the crucial roles of this clade in LMW-DOM remineralization in the Antarctic polynyas.

**Conclusions:**

Genomic reconstruction and in situ gene expression analyses revealed the unique metabolic potential of dominant bacteria of the Antarctic polynya at a finer taxonomic level. The information can be used to predict temporal community succession linked to the availability of substrates derived from the *P. antarctica* bloom. Global warming has resulted in compositional changes in phytoplankton from *P. antarctica* to diatoms, and thus, repeated parallel studies in various polynyas are required to predict global warming-related changes in carbon remineralization.

**Electronic supplementary material:**

The online version of this article (10.1186/s40168-019-0643-4) contains supplementary material, which is available to authorized users.

## Background

Large polynyas developed in the Antarctic coast during the austral summer are ecological hotspots due to extremely productive phytoplankton bloom and intense biogeochemical cycling. In the spring, melting glaciers and sea ice stratify the water column and increase levels of light and nutrients for stimulating the growth of phytoplankton, especially *Phaeocystis antarctica* [1, 2]. *Phaeocystis* is one of the few microalgae that benefits strongly from eutrophication, resulting in almost uni-algal plankton blooms [3, 4]. This alga has a polymorphic life cycle (free-living cells and colonies composed of thousands of cells embedded in a polysaccharidic matrix). Organic matter is released from phytoplankton upon lysis by zooplankton grazing, virus infection, or senescence [5]. In fact, the highest chromophoric dissolved organic matter (DOM) concentrations are frequently observed near the peak of the phytoplankton bloom within polynyas of the Amundsen Sea (the ASPIRE cruise in 2010–2011) [6].

Carbon fixed by phytoplankton in the water column is transferred to higher trophic levels, and finally, is exported from the system to the deep ocean [7]. However, intriguingly, in the Amundsen Sea dominated by *P. antarctica*, most of the organic carbon from primary production does not reach the sediment; instead, it is actively recycled and mineralized in the water column via microbial loop [8–11]. Indeed, higher heterotrophic bacterial production, accounting for ca. 20% of the organic carbon produced by primary production, has been observed in the surface mixed layer of the polynya [9]. These reports indicate that the Amundsen Sea polynya (ASP) is highly inefficient at transporting carbon to the deep sea via biological pump.

Increases in the *p*CO_2_ concentration and water column stratification [12] by global warming may have shifted the phytoplankton community structure from *P. antarctica* to diatoms in the Southern Ocean [12–14]. Diatoms possess relatively faster sinking rate [15, 16]. This makes export flux of organic carbon formed by diatom blooms two times faster than that by *Phaeocystis* blooms [16], which dramatically increases the contribution of diatoms to total export below the photic zone [17].

Bacterial assemblages may be responsible for the enhanced assimilation and remineralization of organic matter from phytoplankton blooms in polynyas. Associations of bacteria with phytoplankton blooms have been reported in polar oceans based on conventional clone library analyses [18]. Using next-generation sequencing approaches with enhanced sequencing depth [19–21], distinct prokaryotic assemblages have been observed at the peak of the *P. antarctica*-dominated bloom in ASP [21, 22]. However, logistical constraints and safety concerns have limited the investigation of the temporal succession of microbial-bloom communities in Antarctic waters—particularly in the decline period (late February) following the peak in phytoplankton activity.

Furthermore, although bacterial community composition in various ocean provinces has been described, ecophysiological and biochemical properties of key bacteria are still hard to predict owing to their low culturability in artificial media. Since free-living bacterioplankton plays a pivotal role in the mineralization of algae-derived organic matter, free-living bacterioplankton was frequently used to explain bacterioplankton succession dynamics during the bloom [23]. Genomic reconstruction techniques from metagenomic sequences can be used to characterize uncultivated lineages of microorganisms. Physiological properties and biochemical pathways can be predicted using genomes reconstructed by de novo assembly and binning from random shotgun genomic libraries that are obtained directly from environmental samples [24–26]. Further, environmental mRNA sequence reads can be mapped to metabolic pathway genes of reconstructed genome bins to evaluate in situ activities [27]. In the present study, nucleic acids harvested from water samples gathered during three cruises were used to reconstruct genomes of key heterotrophic bacteria from a *P. antarctica*-dominant ASP. We also analyzed the expression of genes in the reconstructed genomes using metatranscriptomics. A comparative analysis of samples from different phases of the bloom (peak and decline) and from sea ice-covered ocean provides fundamental insights into the ecology of abundant bacteria associated with phytoplankton blooms in these Antarctic polynyas.

## Methods

### Sampling and geochemical analysis

During three cruises, four water samples from the Amundsen Sea (two each from the peak and decline phases) were gathered from the center of a polynya. The average bloom termination date is February 23 (± 5.38 days) [12]. Satellite-based surface chlorophyll data was obtained from the GlobColour webpage (http://hermes.acri.fr/), which has been constructed from multi ocean color sensors. In addition, a control ocean water sample was gathered from below sea ice by ice core drilling (Additional file 1: Table S1). A rosette water sampler equipped with ten Niskin bottles (10 L) and a conductivity-temperature-depth unit (sensors for Chl-*a* fluorescence, pressure, temperature, and salinity) was deployed. The concentrations of Chl-*a* and inorganic nutrients (nitrate + nitrite, ammonium, phosphate, and silicate) were measured aboard, as described by Kim et al. [1] Briefly, the concentration of Chl-*a* extracted in 90% acetone was measured using a Trilogy fluorometer (Turner Designs, San Jose, CA, USA). The inorganic nutrients were analyzed using a QuAAtro Auto Analyzer (SEAL Analytical, Southampton, UK). Bacterial 16S rRNA gene copies were evaluated by real-time quantitative PCR (qPCR) as described by Kim et al. [21] For qPCR, the Bac518F and Bac786R primer set was used. To evaluate the phytoplankton composition, sampled water was immediately fixed using glutaraldehyde. Filter-harvested phytoplankton cells were stained with a staining solution, and the composition was manually checked using a microscope. The detailed methods are described in Additional file 2: Supplementary methods according to Lee et al. [28]. The depth of the samples was set to the maximum chlorophyll layer.

Free-living bacterioplankton cells were harvested by sequential filtration of 10 L of water through a 1.2-μm pore-sized filter and 0.22-μm pore-sized filter (Supor polyethersulfone, Pall Life Sciences, Ann Arbor, MI, USA). Seawater samples were collected for approximately 1–2 h around midday during each sampling period. Within 30 min of collection, samples were filtered and maintained at under 10 °C. The filters were immediately stored at − 80 °C until further processing in the laboratory.

### Nucleic acid extraction and pyrosequencing

The total nucleic acids were extracted from cells collected on filters with a 0.22-μm pore size using a previously described protocol [29]. Briefly, harvested cells in the filter were grazed with quartz and liquid nitrogen. Grazed cells were lysed with CTAB buffer, and nucleic acids were separated using chloroform. Ethanol precipitation was used to purify and concentrate total nucleic acids. DNA and RNA were separated using the AllPrep DNA/RNA Mini Kit (Qiagen, Valencia, CA, USA) following the manufacturer’s protocols.

For the community analysis, the V1 to V3 regions of the 16S rRNA gene were amplified with the barcoded primer set V1-9F (5′-GAGTTTGATCMTGGCTCAG-3′) and V3-541R (5′-WTTACCGCGGCTGCTGG-3′) [30]. PCR was conducted using the following conditions: 94 °C for 5 min, 30 cycles of denaturation at 94 °C for 30 s, annealing at 55 °C for 45 s, elongation at 72 °C for 60 s, and 72 °C for 5 min for the final extension. The amplified PCR products were purified using the PCR Purification Kit (Cosmogentech, Seoul, Republic of Korea). PCR products for each sample were evenly mixed (0.4 μg per sample), quantified using the NanoDrop Spectrophotometer (Wilmington, DE, USA), and then sequenced by ChunLab Inc. (Seoul, Republic of Korea) using a Roche/454 GS FLX Titanium sequencer. Pyrosequencing data were processed as follows. Chimeric sequences were removed using UCHIME (v. 4.2) [31]. OTUs at the 97% similarity level were obtained using uclust [31]. A taxonomic classification was assigned to each read using Greengenes as a reference database [32]. These steps were performed using the pick_de_novo_otus.py script in QIIME (ver. 1.9.1). Unassigned reads that showed no match to sequences in the Greengenes database were manually reassigned against NCBI Microbial 16S rRNA gene database using BLASTn (cutoff, *e* value 1 × E−5). Diversity and species richness indices were calculated using the alpha_diversity.py script with 5456 reads subsampled from each sample. Neighbor-joining trees were constructed using representative sequences of each OTU selected by QIIME. These sequences were compared against the NCBI nucleotide database and the EzBioCloud database to select the nearest neighbors. Selected sequences were aligned and edited using BioEdit, and a tree was reconstructed using MEGA 7.0 [33] with the Kimura 2-parameter model with 1000 bootstrap replicates. Additionally, previous pyrosequencing data obtained during the 2010/2011 cruise [21] were reanalyzed using QIIME to compare bacterial communities.

### Statistical analysis of environmental factors and the microbial community

To identify the relationships between major bacterial groups and the environmental parameters, a non-metric multidimensional scaling (NMDS) analysis was performed using R with the vegan package (v. 2.4).

### Metagenome sequencing, assembly, and annotation

Three genomic DNAs obtained above (marked in Additional file 1: Table S1; where PK indicates the peak phase of the bloom, DC indicates the declining phase, and SI indicates the ocean sample under sea ice) were used to construct Illumina sequencing libraries with target insert sizes of 300 and 5000 bp using the TruSeq PCR Free Kit and Nextera Mate Pair Kit, respectively. Fragments of libraries were used for paired-end and mate-pair sequencing using the Illumina Hiseq2000 according to the manufacturer’s instructions at Macrogen (Seoul, Republic of Korea). Raw reads were trimmed using Sickle with default settings (https://github.com/najoshi/sickle/). Each paired-end read set was assembled using the IDBA-UD (v. 1.1.1) assembler since it provided the best results. The quality of assembly was determined by using N50. Default settings in IDBA_UD were used since those are normally suitable for most metagenome datasets (https://ggkbase-help.berkeley.edu/overview/data-preparation-metagenome/) [34].Scaffolding was performed by SSPACE (v. 2.1) using preassembled contigs from each sample with mate-pair reads [35]. For taxonomic classification of metagenome and metatranscriptome reads, Centrifuge (v. 1.0.4) was used against NT database (supported by Centrifuge) [36].

For scaffolds of ≥ 1 kb, putative genes were predicted using MetaGeneAnnotator [37]. Functions of the protein sequences were predicted using BLASTp similarity searches against the NCBI NR database, KEGG, COG, and TIGR (*e* value cutoff; 1 × E−5). The rRNAs and tRNAs were identified using RNAmmer [38] and tRNAscan-SE [39], respectively. Domain information was obtained using the Pfam database (pfam_scan.pl, default parameters). TonB-dependent transporters (TBDTs) were classified as described by Tang et al. [40]. For the classification of ABC transporters and peptidases, putative genes were compared against the TCDB [41] and MEROPS databases [42] using BLASTp (*e* value cutoff; 1 × E−5), respectively. Signal peptides were predicted for the determination of extracellular proteins using SignalP version 4.1 [43].

### Binning and phylogenetic analysis

The binning was performed with SI metagenomic scaffolds using the differential coverage plotting method in combination with the tetranucleotide frequency, as described by Albertsen et al. [44] based on R script (http://madsalbertsen.github.io/multi-metagenome//). Bowtie2 (v. 2.1.0) and SAMtools (v. 1.9) were used to calculate scaffold coverages [45, 46]. The coverage value was calculated according to Lander and Waterman [47] as *LN*/*G*, where *L* is the read length, *N* is the number of mapped reads, and *G* is the scaffold length (excluding *N*s). After binning, unmatched scaffolds to the bin were manually removed according to a taxonomic analysis of scaffolds. The taxonomy of scaffold was determined based on an assignment of the majority of its genes to a specific taxon using BLASTp against the NR database. To estimate the genome completeness of reconstructed bins, CheckM (v. 1.0.5) was used [48]. Using criteria (completeness and contamination) evaluated using CheckM, final bins were selected for further analysis. For the taxonomic classification of genomes, rRNA (*rpoB*; in case of GM5, *recA* was used because this bin lacked the *rpoB* gene) was used. Single marker genes were analyzed using BLASTp against the NR database. Hits for 16S rRNA genes were extracted from the total reads using BLASTn (alignment length ≥ 100 and *e* value ≤ 0.001) for comparisons of pyrosequencing data [49].

### Metatranscriptomic analysis

The TruSeq RNA Kit was used to generate the cDNA template library from extracted RNA, and transcript reads were obtained by Illumina paired-end Hiseq2000 sequencing according to the manufacturer’s instructions at Macrogen. Raw transcript reads were preprocessed using the FASTX-Toolkit (v. 0.0.14) for quality filtering (minimum quality score, 20; minimum percent of bases that must have a quality score exceeding 20, 90; http://hannonlab.cshl.edu/fastx_toolkit/). To remove rRNA reads, reads with matches in the SILVA database (SSU and LSU ver. 127) [50] and 5S rRNA database [51] were removed using Bowtie2 and SAMtools. Reads mapped to eukaryotic and viral genomes were removed using Centrifuge (v. 1.0.4). For the transcriptomic analysis, predicted bacterial scaffolds among assembled scaffolds exceeding 1 kb obtained from SI were also used as a custom nucleotide database. Non-rRNA cDNA reads from three samples were mapped against the predicted gene of the custom nucleotide database using Bowtie2 (under default parameters), and assigned reads were counted using SAMtools. Unmapped reads were aligned against UniRef90 using DIAMOND blastx search (query cover 90, *e* value 1, according to HUMAnN2) [52, 53]. Finally, mapped reads to metagenome and UniRef90 were used for mRNA normalization. Expression levels of individual genes were obtained using TPM value (transcripts per kilobase million).

### Correlation between DNA-TPM and mRNA-TPM

The normality of the population distribution was evaluated using the Shapiro–Wilk test for samples of mRNA-TPM. As the data were not normally distributed, the Spearman rank correlation was determined using R (vegan package, v. 2.4) to determine correlations between PK-mRNA-TPM:DC-mRNA-TPM for each sample.

## Results

### Oceanographic properties

Samples were obtained from phytoplankton blooms of ASP during three cruises (2010–2011, 2011–2012, and 2013–2014) in austral summer. During the 2010–2011 and 2013–2014 cruises, samples from the peak phase of the bloom were obtained. As indicated in Additional file 3: Figure S1, the expedition in 2011–2012 was scheduled to collect two samples in the decline phase of the phytoplankton bloom (Feb. 14 and 29 in 2012). A sample of sea ice-covered ocean water was included for comparison. *P. antarctica* was the predominant primary producer at the polynya stations, whereas diatoms predominated at the sea ice station (Table 1).

**Table 1:** Description of the location and oceanographic properties of sampling sites

	Peak-1	Declining-1^b^	Declining-2	Peak-2^b^	Sea ice^b^
Sampling date	2010/2011 cruise; 07 Jan 2011	2011/2012 cruise; 14 Feb 2012	2011/2012 cruise; 29 Feb 2012	2013/2014 cruise; 03 Jan 2014	2010/2011 cruise; 05 Jan 2011
Sampling point^a^ (depth)	Polynya (30 m)	Polynya (20 m)	Polynya (25 m)	Polynya (20 m)	Under sea ice (2 m)
Abundance of bacteria (×10^6^ 16S rRNA gene copies/mL)	2	25	9.7	7.2	1.4
NO_2_^-^ + NO_3_^-^ (μmol/L)	15.0	9.6	16.6	14.9	26.6
NH_4_^+^ (μmol/L)	0.5	0.3	0.6	0.03	1.6
PO_4_^2-^ (μmol/L)	N/D	1.0	1.4	1.4	N/D
SiO_2_ (μmol/L)	79.4	74.7	77.4	84.8	76.7
Chl-a (mg/L)	9.5	5.9	2.4	5.8	1.0
Phytoplankton composition (μg C/L)					
*Phaeocystis antarctica*	279.8	174.0	38.5	255.3	6.0
Autotrophic picoplankton	2.4	10.0	1.7	0.02	3.7
Autotrophic flagellates	6.7	2.8	4.4	3.6	0.9
*Diatom*	83.0	16.6	15.6	7.9	67.0
Total carbon (μg C/L)	367.6	203.3	60.1	266.8	77.6
Carbon to Chl-*a*	38.69	34.6	25.09	46	81.7

^a^GPS position: polynya center station, 73.30 (LAT) -113.02 (LONG); sea ice station 72.30 (LAT), -112.44 (LONG)

^b^Used for metagenomics and metatranscriptomic analyses

### Bacterial community composition and dynamics

Based on an analysis of 16S rRNA gene sequences, alpha diversity indices in the decline phase were greater than those during the peak phase of the bloom and in the sea ice-covered ocean (Additional file 1: Table S1). *Bacteroidetes* and *Proteobacteria* were the most abundant phyla in all samples (Additional file 1: Table S1). Reads of 16S rRNA gene sequences of *Polaribacter*, the gammaproteobacterium Ant4D3 (*Oceanospirillaceae*), and *Pelagibacter* were abundant at ASP. *Polaribacter* sequences were particularly dominant (37.4–50.5% of total sequences) in the peak phase of the bloom. Despite differences between bloom phases, the major OTUs assigned to *Polaribacter*, SAR92, Ant4D3, and SAR11 were nearly identical (Additional file 3: Figure S2). The taxonomic distributions are described in detail in Additional file 1: Table S1.

A NMDS analysis of bacterial communities showed that the communities at the peak phase clustered with each other but were distinct from those at the decline phase or in the sea ice-covered ocean, although they were collected in different years (Additional file 3: Figure S3). The bacterial communities in the two different decline-phase samples clustered strongly together, despite differences in various factors, such as *P. antarctica* abundance, total algal carbon, and total bacterial abundance. The bacterial communities in the sea ice-covered oceans were variable but were distinct from those in polynya samples. The major environmental parameters influencing bacterial community structure were Chl-*a* and nitrite + nitrate concentration and depth, and the samples of peak phase were positively related to the Chl-*a* concentration.

### Genome reconstruction

To determine the metabolic potential and activities of key bacteria in the different bloom phases, samples at the peak phase (2013–2014; PK) and the decline phase (first sample in 2011–2012; DC) as well as a sample from the sea ice-covered ocean (SI) were selected for genomic reconstruction and transcriptomic analyses. Using the coverage plots of scaffolds assembled from raw sequence reads, genome binning was performed (Additional file 3: Figure S4). Clusters of scaffolds were selected with a threshold of > 100× average coverage in at least one dimension combined with the tetranucleotide frequency (Additional file 3: Figure S4). For genome reconstruction, 12 bins were used. A CheckM analysis indicated that the 12 reconstructed genomes ranged from partially to nearly complete (between 53.9 and 93.6%, except for BC5 (18%)). Using typical phylogenetic marker genes (Additional file 4: Table S2), the phylogenetic positions and contamination were evaluated for the 12 genomes, as shown in Table 2 (see Additional file 1: Table S1). Overall, representative bacterial genomes of dominant clades in the analysis of 16S rRNA gene could be obtained.

**Table 2:** Features of the twelve reconstructed genomes (genome bins) identified by an analysis of metagenomic sequences derived from DNA extracted from Antarctic waters

Property	BC1_Pol	BC2	BC3	BC4	BC5_Pol	GM1_Ant	GM2_Ant	GM3	GM4_SAR92	GM5	GM6_SUP05	AL1_pel
Completeness	87.3	53.9	93.6	77.5	18.4	73.8	79.8	57.6	54.7	54.4	55.8	78.4
Contamination	4.2	0.2	0.1	0.4	0	0.9	1.6	1.1	10.6	2.8	6.0	98.9
Heterogeneity	89.5	0	0	100	0	100	80	14.3	73.7	93.3	79.0	72.6
Total scaffold length (contig length) (Mb)	3.0 (2.6)	2.1 (1.4)	2.8 (2.8)	1.8 (1.7)	2.0 (1.1)	2.1 (1.5)	2.3 (1.7)	3.8 (2.0)	3.6 (1.8)	1.5 (0.9)	2.9 (1.4)	11.3 (4.1)
Number of scaffolds	154	297	13	29	280	36	68	200	490	127	281	1931
N50	60,958	15,726	354,639	126,418	11,663	138,520	96,141	34,087	11,881	20,307	16,782	7,407
Phylogenetic affiliation	*Polaribacter*	*Flavobacterium*	*Flavobacteraceae*	*Ulvibacter*	*Polaribacter*	Ant4D3clade	Ant4D3Clade	*Gammaproteobacteria*	SAR92 clade	*Methylophaga*	SUP05 clade	*Pelagibacter*
Key genes for carbon assimilation (#)												
ABC transporter^a^	24	10	18	21	11	73	97	100	36	8	147	255
TBDT	50	28	24	19	32	1	0	5	34	6	2	0
SusD^b^	10	3	1	2	5	0	0	0	0	0	0	0
TRAP^b^	0	0	0	0	0	12	16	12	3	0	5	80
GH^b^	27	9	4	10	13	1	0	5	8	0	4	16
Peptidase	131	78	107	77	58	62	69	80	88	45	67	238
Sulfatase^b^	10	2	0	2	10	1	2	0	4	0	1	0

^a^Involved in substrate uptake

^b^Based on the Pfam classification

### Rank abundances of reconstructed genomes

**Fig. 1:**
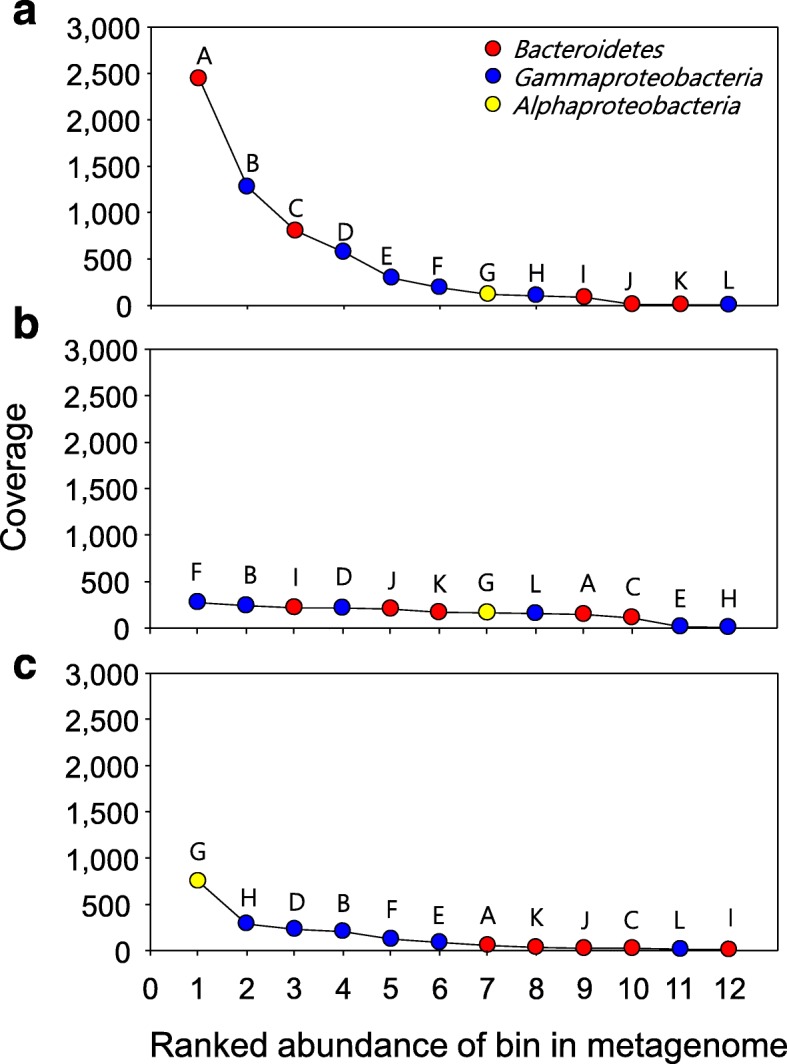
Ranked abundance of selected bins. The coverage values of DNA reads (**a**–**c**) were determined by the average number of reads that align to scaffolds. DNA reads were used from each sample in PK (**a**), DC (**b**), and SI (**c**). A, BC1_Pol; B, GM1_Ant; C, BC5_Pol; D, GM4_SAR92; E, GM5; F, GM2_Ant; G, AL1_Pel; H, GM6_SUP05; I, BC2; J, BC3; K, BC4; L, GM3

The coverage of metagenome-assembled genomes can be used to estimate genome abundance at each phase of the bloom (Fig. 1). As expected from the community composition analysis (Additional file 1: Table S1), DNA read coverages of genomes of *Polaribacter* (BC1_Pol and BC5_Pol) were highest at the peak bloom (ca. 2400× and 800×, respectively), indicating that BC1_Pol and BC5_Pol are the key ecotypes of *Polaribacter* for understanding carbon remineralization in the peak phase (Fig. 1a). The coverage values for BC1_Pol and BC5_Pol diminished during the transition to the decline phases of the bloom, while the coverages of other *Bacteroidetes*, such as BC2, BC3, and BC4, increased. In *Gammaproteobacteria*, the abundance of GM1_Ant and GM4_SAR92 decreased from the peak to the decline phase, while the abundance of GM2_Ant and GM3 increased in the decline phase. Eventually, most of the genomes belonging to *Bacteroidetes*, *Gammaproteobacteria*, and *Alphaproteobacteria* had comparable coverages from 100× to 270× in the decline phase (except GM5 and GM6_SUP05) (Fig. 1b). The highest coverage for AL1_Pel was observed in the sea ice-covered ocean sample (750×, Fig. 1c), with much lower coverages during the peak and decline phases (116× and 163×, respectively). To examine possible reasons for the difference in abundance between pyrosequencing and metagenomic reads-based coverage, read mapping was performed using the genomes of *Pelagibacter* (*Pelagibacter ubique* HTCC 1062, *Pelagibacter* sp. HTCC7211, and *Pelagibacter* sp. IMCC9063) as a database. However, the total mapping read count was lower than those of AL1_Pel (Additional file 1: Table S3). Further, as shown in Additional file 1: Table S4, rRNA gene reads of *Alphaproteobacteria* (including *Pelagibacter*) were lower than those in pyrosequencing results. Thus, we assume that the PCR-biased community analysis overestimated *Pelagibacter* in the Southern Ocean since the same DNA was used for both pyrosequencing and metagenome sequencing [54].

### mRNA transcript abundance

mRNA reads from PK, DC, and SI were classified as shown in Additional file 1: Table S5. Among non-rRNA (mRNA) reads, 66.2%, 42.1%, and 63.1% were matched to the total metagenomic scaffolds and uniref90 database for PK, DC, and SI, respectively (Additional file 1: Table S6). In the peak phase, approximately 25.1% and 6.4% of mRNA transcripts were assigned to genes of BC1_Pol and GM1_Ant, respectively, suggesting their high abundances. In SI, 3.4% of total bacterial mRNA reads matched to genes of AL1_Pel.

Pairwise comparisons of the expression levels of all genes (mRNA-TPM:mRNA-TPM) in PK and DC showed a low overall correlation (*p* = 0.16). However, in an individual genome bin-based pairwise comparison of gene expression between PK and DC, a high correlation of gene expression was observed, with regression slopes ranging from 0.44 to 0.90 (Additional file 3: Figure S5; Additional file 1: Table S7). The strong correlation was further supported by the low average fold changes in transcripts in most of the bins between PK and DC (Additional file 1: Table S8).

### Traits overrepresented in each phase

To investigate the specialized metabolic potential for biogeochemical cycling, gene transcripts abundant during each phase of the bloom were analyzed using reconstructed genome-based approaches. Pairwise comparisons of the expression (mRNA-TPM: mRNA-TPM) of genes involved in biogeochemical processes indicated that gene transcripts overrepresented in PK and DC belong to the taxa with the highest abundance in PK and DC in the metagenome, respectively (Additional file 3: Figure S6a). The expression of distinct repertoires of genes for transporters, the glycoside hydrolase (GH) family, and the assimilation of compatible solutes (Additional file 3: Figure S6b–e) were overrepresented at each phase and were further investigated.

### Transporters

**Fig. 2:**
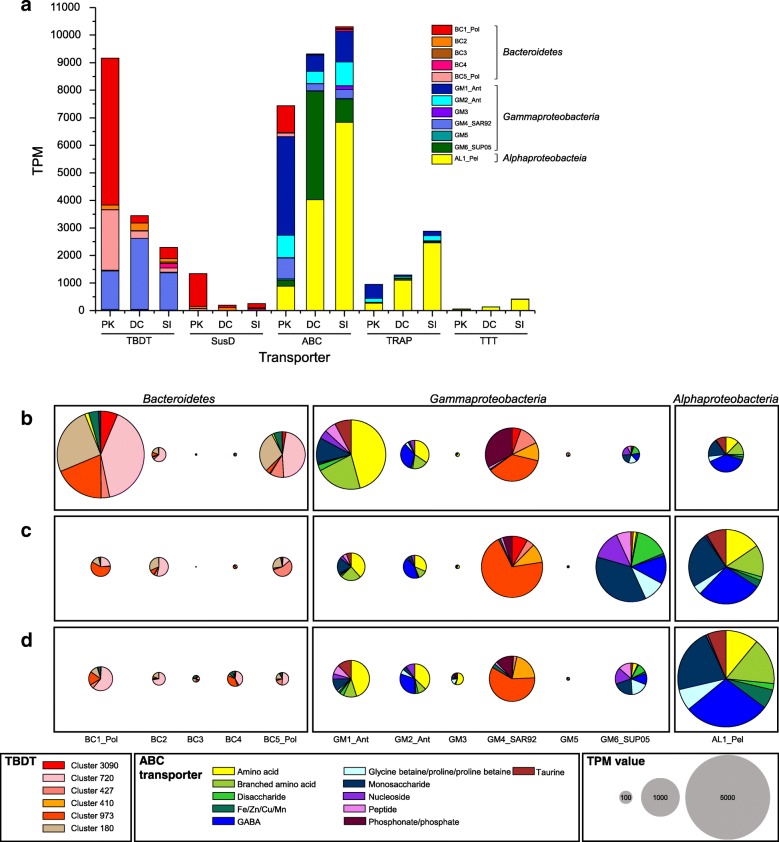
Relative gene expression levels based on mRNA-TPM of transporters. **a** TonB-dependent transporters (TBDT), SusD-family proteins (SusD), ABC (ATP-dependent binding cassette), tripartite ATP-independent periplasmic (TRAP) transporters, and tripartite tricarboxylate transporter (TTT) in selected bins at PK, DC, and SI. Functionally classified TBDT and ABC transporters in **b** PK (top), **c** DC (mid), and **d** SI (bottom). Transcript values of less than 200 TPM value in all bins are not shown

Transcripts assigned to putative transporter genes in selected bins were highly represented in our metatranscriptome (2.3%, 1.0%, and 1.8% of total mRNA obtained from PK, DC, and SI, respectively; Fig. 2a). The phylogenetic position of the metagenome-assembled genome corresponded to distinct transporter types (Additional file 1: Table S9). TBDT-like transcripts in BC1_Pol and BC5_Pol bins were prominent (Fig. 2a). Transcripts of putative TBDT genes involved in the uptake of various digested biopolymers (cluster 720 and 3090) were abundant in *Polaribacter* bins (BC1_Pol and BC5_Pol) (Fig. 2b). Putative genes encoding SusD, a key protein required for glycan binding and uptake by TBDT-mediated transporters [55], were enriched in *Polaribacter* bins (*n* = 10 and 5 in BC1_Pol and BC5_Pol, respectively (Table 2; Additional file 1: Table S9) and were highly expressed (Fig. 2a). Genes encoding transporters for vitamin B_12_ (cluster 973) and thiamin (cluster 180) in BC1_Pol were highly expressed at the peak phase (Fig. 2b). GM1_Ant and GM2_Ant may be potential sources of vitamin B_12_ (see Additional file 1: Table S10).

Although GM4_SAR92 belongs to *Gammaproteobacteria*, putative genes for TBDTs were enriched (Additional file 1: Table S9) and transcripts assigned to the putative genes were abundant. In particular, transcripts assigned to TBDT cluster 427 for the uptake of arabinose, a major component of the saccharide pools of *Phaeocystis* cells [15], were observed at the peak phase (Fig. 2b). In addition, putative genes for TBDTs of cluster 973 and 410 in GM4_SAR92 involved in the transport of vitamin B_12_ and various cofactors (especially iron), respectively, were similarly expressed in all three samples.

Putative genes encoding ABC transporters specific for the uptake of amino acids, branched amino acids, peptides, and monosaccharides were abundant in GM1_Ant and GM2_Ant (*n* = 73 and *n* = 97, respectively), and transcripts assigned to these putative genes were abundant in the peak phase (Table 2 and Fig. 2b). Transcripts of putative genes for the ABC transporter for glycerol uptake were also prominent in GM2_Ant (Fig. 2b). Further, putative TRAP genes (in particular, for mannitol uptake) were enriched (*n* = 12 and *n* = 16, respectively) in GM1_Ant and GM2_Ant, with high recruitment of transcripts in the peak phase (Fig. 2). Glycerol and mannitol are used as osmolytes [56] and might be widely available substrates in polynya blooms. Putative genes encoding mono- and disaccharide, peptide, and glycine betaine (GB) ABC transporters were abundant in GAM6_SUP05 and highly expressed in the decline phase (Fig. 2c). High abundances of transcripts for ABC and TRAP transporters in AL1_Pel for low molecular weight (LMW)-DOM, such as amino acids, taurine, mono- and disaccharides, were observed in the sea ice-covered ocean (Fig. 2d).

### Utilization of polysaccharides

Putative genes in the GH family were abundant (*n* = 27) in BC1_Pol (Table 2), i.e., there were more than four copies of GH16, GH20, and GH92 for the degradation of laminarin, chitin, and mannose-containing polysaccharides, respectively. Many of these putative genes were co-localized with TBDTs and SusD genes in five putative polysaccharide utilization loci (PUL). In contrast to BC1_Pol, BC5_Pol contained six of ten putative sulfatase genes residing in PUL associated with the potential for sulfated polysaccharide assimilation.

**Fig. 3:**
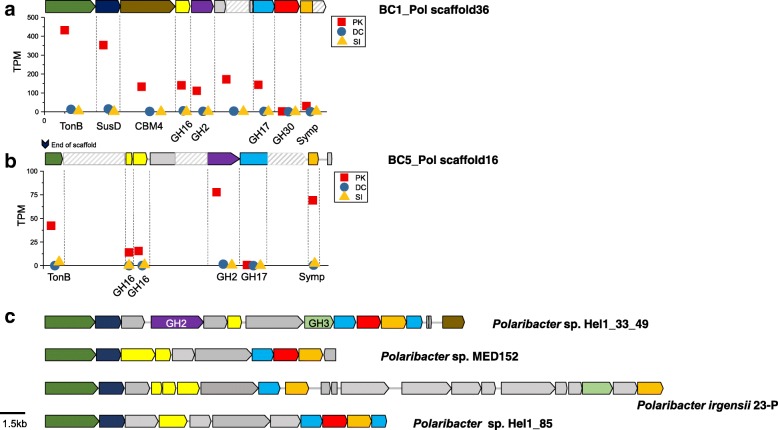
Representative polysaccharide utilization loci (PUL) related to laminarin utilization in *Polaribacter* bins and reference *Polaribacter* genomes. **a**, **b** Expression levels of a gene in the PUL of BC1_Pol and BC5_Pol, respectively. Gene expression levels were determined by TPM value. For comparison, **c** shows similar PULs of *Polaribacter* spp. including the laminarin-induced PULs in the genomes of *Polaribacter* sp. Hel1_33_49 and Hel1_85 obtained from a diatom bloom [82]. TBDT, TonB-dependent transporter; SusD, SusD-family protein; CBM4; carbohydrate binding module; GH16, glycoside hydrolase 16 (laminarinase); GH2, glycoside hydrolase 2; GH3, glycoside hydrolase 3; GH17, glycoside hydrolase 17; GH30, glycoside hydrolase 30; Symp, sugar:H^+^ symporter

**Fig. 4:**
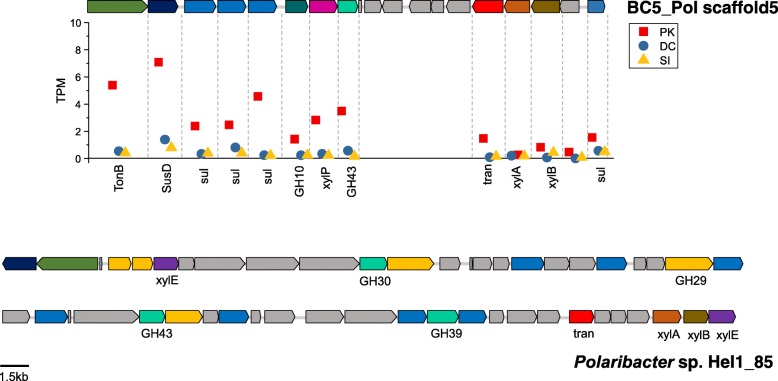
A PUL related to xylan utilization in BC5_Pol with expression levels of genes. A cluster for xylose assimilation in *Polaribacter* sp. Hel1_85 is shown for comparison. Normalization for the estimation of the relative abundance was conducted as described in the legend of Fig. 3. TonB, TonB-dependent transporter; SusD, SusD-family protein; sul, sulfatase; GH10, glycoside hydrolase 10 (endo-1,4-beta-xylanase); GH43, glycoside hydrolase 43 (xylan 1,4 xylosidase); xylP and xylE, xylose:H+ symporter; tran, putative transporter; xylA, xylose isomerase; xylB. xylulose kinase; GH30, glycoside hydrolase 30 (beta-xylosidase); GH29, glycoside hydrolase 29 (alpha-l-fucosidase); GH39, glycoside hydrolase 39 (xylan 1,4 xylosidase)

Transcripts from putative genes encoding GHs (GH2, GH16, GH17, and GH30) in a PUL for laminarin degradation were abundant in BC1_Pol in the peak phase (Additional file 3: Figure S6c; Fig. 3). A xylan utilization-related PUL containing various putative genes for xylan degradation and assimilation was observed in BC5_Pol (Fig. 4). Among known *Polaribacter* genomes, the genetic potential for xylan degradation (GH10, endo-1,4-beta-xylanase) was observed only in BC5_Pol. Interestingly, PUL was not observed in BC3, and BC4 bins and the abundances of transcripts assigned to GHs in the BC3 and BC4 bins were very low0 (Additional file 3: Figure S6c). Although PUL was not observed, transcripts from putative genes encoding GHs, such as glucan β-1,4-glucosidase (GH3) and laminarinase (GH16), were detected in GM4_SAR92 (Additional file 3: Figure S7) in addition to the high representation of TBDT genes (Fig. 2).

### Assimilation of dimethylpropionate

Putative genes for dimethylpropionate(DMSP) demethylase (*dmdA*) were widely present and expressed in the major bacteria in the PK, DC, and SI samples, i.e., GM1_Ant and GM4_SAR92 (Additional file 3: Figure S8a). While *dmdA* of GM1_Ant was highly expressed at PK, that of GM4_SAR92 was highly expressed in all three samples. Putative genes related to the assimilation of 3-methylmercaptopropionate (MMPA) were co-localized with DMSP demethylase in GM1_Ant, GM2_Ant, and GM3 (Additional file 3: Figure S8b). Assimilation of glycine betaine and other compatible solutes is described in the Additional file 2: Supplementary results.

## Discussion

Phytoplankton blooms in Antarctic polynyas are accompanied by various bacterial taxa, as revealed by 16S rRNA gene analyses [21]. In order to investigate bacterial plankton succession dynamics during the bloom, we used samples for two phases, namely the peak and decline of bloom collected over different years. Due to the unique nature of blooms in ASP (i.e., a short period of opening in the sea ice, low temperature, the abundance of *P. antarctica*-derived organic matter), inter-annual variation in biodiversity may be limited, and thus, bacterioplankton dynamics likely exhibit recurrent patterns. Similarly, repeated patterns have been observed in the North Sea among specific bacterial clades of four consecutive blooms, despite some inter-annual variation in physicochemical conditions [23]. Moreover, the dominance of *Polaribacter* at the peak of the bloom has been observed evident in previous studies of ASP [21, 57]. The obligate psychrophilic nature of *Polaribacter* spp. among members of *Bacteroidetes* isolated from Southern Ocean including ASP [58] may contribute to their adaptation to blooms of ASP [59]. The high ANI value and conserved synteny between fosmid clone Ant4D3 [60] and GM1_Ant, which was dominant in all phases of the polynya bloom (see Additional file 3: Figure S9), support the recurring pattern of the most well-adapted clades in the Southern Ocean.

For adaptation to the polynya blooms, each dominant bacterial clade may harbor distinct metabolic properties. Our genome reconstruction and metatranscriptomic analysis provide relevant information on the major taxa responsible for DOM utilization in the ASP. *Polaribacter* and SAR92 clades had the potential to utilize HMW-DOC, while the Ant4D3, SAR92, and SUP05 clades had the potential to utilize LMW-DOC. Further fine-scale taxonomic (ecotype) differences could also be observed.

In contrast to diatom-dominated spring bloom in the North Sea, where the expression of GHs most likely allowed diverse members of *Bacteroidetes* to decompose algal biopolymers [61], *Polaribacter* was the single predominant clade associated with the degradation of organic matter derived from *P. antarctica* at the peak bloom in the ASP (Table 1). The direct release of LMW-DOM by growing *P. antarctica* cells is low [62–64], and most DOM from intact *P. antarctica* cells is composed of polysaccharides. This corresponds with the predominance of novel *Polaribacter* ecotypes, BC1_Pol and BC5_Pol.

Chrysolaminarin, a β-1,3-glucan, laminarinase (GH16)-specific substrate, is considered one of the most abundant polysaccharides in *Phaeocystis* blooms [15]. BC1_Pol might be specialized to degrade chrysolaminarin with high expression of a laminarinase (GH16) gene in PUL. Arabinose and xylose are predominant in the carbohydrate composition of *Phaeocystis* [15, 65], which is a crucial distinction from diatoms [66]. Thus, pentose-containing polysaccharides might be widely available substrates during *P. antarctica* blooms, and BC5_Pol with the genetic potential for xylan degradation (GH10, endo-1,4-beta-xylanase) may be specialized for the utilization of these polysaccharides. Despite the high average nucleotide identity (ANI 91.8%) between BC1_Pol and BC5_Pol, our results indicate differentiation in the utilization of polysaccharides (e.g., chrysolaminarin and xylan with GH16 and GH10, respectively; Fig. 3 and Fig. 4), which are significant polymers in *P. antarctica* [15], using a distinct set of GHs. Similar to observations by Teeling et al. [23], frequencies of CAZymes of BC1_Pol and BC5_Pol in metagenomes and metatranscriptomes were significantly decreased as blooms subsided in DC. Detection of transcripts from putative genes encoding GHs in addition to the high representation of TBDT genes in GM4_SAR92 indicates a potential role of GM4_SAR92 in the degradation and assimilation of algae-derived polysaccharides, as previously suggested in a diatom bloom [27], which is a key distinction from other members of *Gammaproteobacteria*.

*Phaeocystis* accumulates large amounts of dimethylpropionate (DMSP) (~ 150 mM in cells) as a compatible solute, which can be released from decaying *P. antarctica* cells [67, 68]. One of the common traits of the abundant Ant4D3 and SAR92 clades in the Amundsen Sea was the metabolic potential of C1 compounds associated with the assimilation of these compatible solutes. DMSP can be converted to a highly reactive volatile sulfur species, methanethiol (MeSH), by DMSP demethylase (DmdA) (see Additional file 3: Figure S8c). MeSH is likely to remain in the ocean surface and is readily used by marine bacteria as a carbon and sulfur source [69]. DMSP lyase (DddD) and CoA transferase (DddD and DddL, − W and − Y) catalyze key biochemical steps in the conversion of DMSP to dimethyl sulfide (DMS), an important climate change gas [70]. Remarkably, representative genes encoding DMSP lyase and CoA transferase were not observed in the total assembled scaffolds. Since significant concentrations of DMS were observed at these study sites [71], DMSP lyase of *Phaeocystis* [72] or an unknown pathway might be involved in DMS production in the polynya.

Unique LMW-DOM-assimilating bacterial assemblages in the decline phase in the Amundsen Sea are dictated by gene expression in the SUP05, SAR92, and Ant4D3 clades of *Gammaproteobacteria* (Additional file 3: Figure S6d), while the RCA (*Roseobacter* clade-affiliated) cluster of *Alphaproteobacteria* assimilates LMW-DOM in spring algal blooms in other oceans [73–75]. A comparative genomic analysis indicated that the metabolic properties of the abundant clades of *Gammaproteobacteria* largely overlap with those of the RCA cluster, e.g., amino acid and C1 compound (e.g., DMSP) assimilation and cobalamin synthesis (Additional file 5: Table S11). Additionally, the differentiation of the clades of *Gammaproteobacteria* from the RCA cluster can be observed in the glyoxylate shunt, proteorhodopsin synthesis, CO oxidation, and urea utilization (Additional file 1: Table S10). Isocitrate lyase of GM6_SUP05 and GM1_Ant was highly expressed (ranked 229th and 137th in the expression in PK and DC, respectively (Additional file 1: Table S12). At polynyas with iron limitation, cells with the glyoxylate shunt can use fewer hemeproteins and make fewer electron carriers to decrease oxidative stress [76]. Genes for CODH were only observed in RCA clades for which iron is required as an essential cofactor. Insufficient ATP or proton motive force can be supplemented by proteorhodopsin, which is an abundant gene transcript in the SUP05, SAR92, and Ant4D3 clades in both PK and DC (Additional file 3: Figure S6e). These genomic features may contribute to the dominance of gammaproteobacterial clades in *Phaeocystis-*dominant Antarctic blooms in which iron is depleted [77]. The possible depletion of iron was supported by the high expression of genes encoding TBDT and ABC transporters involved in iron transport in most of the genomes (Fig. 2).

The polynya is a biogeochemical hotspot where high carbon biomass reached 10 mg C 1^−1^is sequestered via biological pump [78]. Surprisingly, an exceptionally low amount of fixed carbon is exported to the ASP [8]. The low recalcitrance of the DOM derived from the slowly sinking *Phaeocystis* is likely to be associated with rapid mineralization of the organic matter in the water column [79, 80]. Laboratory studies have revealed that carbohydrates derived from *Phaeocystis* were readily degraded by heterotrophic bacteria [81]. Together, *P. antarctica*-dominated bloom may contribute relatively uniform and readily degradable organic matter, which can be efficiently degraded by a few specialized bacterial clades, contributing to high rates of remineralization of fixed carbon.

## Conclusions

In this study, genomic reconstruction and in situ gene expression analyses were used to characterize the metabolic contributions of dominant bacteria native to the ASP to understand carbon remineralization processes. This analysis allowed us to evaluate the major findings of previous studies of the Southern Ocean at a finer taxonomic level. The metabolic potential of dominant bacterial clades of *Bacteroidetes* and *Gammaproteobacteria* can be used to predict temporal community succession linked to the availability of substrates derived from phases of the *P. antarctica* bloom. Global warming has resulted in compositional changes in phytoplankton from *Phaeocystis* to diatoms in Antarctic polynyas [12–14]. This change might be accompanied by bacterioplankton community changes, which may affect carbon remineralization and thus carbon sequestration. Repeated parallel studies of the carbon remineralization potential of bacterioplankton in diatom-dominated polynyas are required to predict global warming-related changes in biogeochemical cycles in polynyas.

## Additional files

Additional file 1: **Table S1.** Diversity and abundance of bacterial 16S rRNA gene sequences obtained by the pyrosequencing of PCR amplicons in this study. Taxa with frequencies of < 1% were omitted from all samples. N/D, not detected. **Table S3.** DNA reads mapped to AL1_Pel and three *Pelagibacter* genomes. **Table S4.** Abundance (phylum level) of 16S rRNA gene among DNA reads. tr, < 0.1%; N/D, not detected. **Table S5.** Raw read classification based on NT database using Centrifuge. **Table S6.** Summary of metagenome and metatranscriptome data. **Table S7.** Spearman correlation coefficients for genes in the PK-mRNA-TPM and DC-mRNA-TPM datasets. **Table S8.** Bloom phase-specific gene expression and average fold changes in expression in 12 genome bins. **Table S9.** Summary of genes encoding representative transporters (TBDT, ABC, and TRAP) and SusD from 12 genomes. **Table S10.** Comparison of selected genes and pathways in GM1_Ant, GM2_Ant, GM4_SAR92, GM6_SUP05, and *Roseobacter* clades. **Table S12.** Genes in the pathway for vitamin B_12_ biosynthesis in GM1_Ant, GM2_Ant, and GM4_SAR92. (DOCX 73 kb)

Additional file 2: Supplementary Results and Methods. (DOCX 939 kb)

Additional file 3: **Figure S1.** A) Satellite-based surface chlorophyll-a concentrations (mg/m^3^) and photosynthetically available radiance (PAR) in the polynya of the Amundsen Sea during the sampling cruises. B) Detailed satellite-based surface chlorophyll-a concentrations (mg/m^3^) and sampling day of this study. GPS position polynya center station, 73.25–73.75S, 114.25–113.75 W. The data constructed from multi ocean color sensors were obtained from the Globcolour webpage (http://hermes.acri.fr/). **Figure S2.** Neighbor-joining tree of the most abundant OTUs in a) *Polaribacter*, b) *Oceanospirillaceae*, c) SAR92, and d) SAR11 from PK, DC, and SI. The representative sequences of each OTU selected by QIIME are highlighted in bold and coded as follows: OTU N (=OTU number), site (PK, DC, or SI), and percentage of the total read number. Bootstrap values of ≥ 50% are shown. **Figure S3.** NMDS plot showing relationships between bacterial compositions and environmental variables. Bray–Curtis stress 0.117. *indicates *p* < 0.05 for the environmental parameter. Samples from station 1 to station 26 from Kim et al. [19] were reanalyzed. Samples from the polynya center in this study are represented as filled squares; green indicates the peak phase of the bloom, and red indicates the declining phase of the bloom. Filled triangles represent samples obtained under sea ice. Samples from the previous study are represented as empty squares for the polynya center (station 13), empty diamonds for the polynya margin (station 8), empty pentagons for the polynya ice shelf (station 11), empty circles for the open ocean (station 1), and empty triangles for water under sea ice (station 26). **Figure S4.** Differential coverage plot of scaffolds for DNA reads obtained from a) PK, b) DC, and c, d) SI. Samples used for coverage calculation are marked on the *X*-axis (decline phase of the bloom) and *Y*-axis (peak phase of the bloom). Summary of a principal component analysis of the tetranucleotide frequencies of scaffolds selected from differential coverage plot: e) BC1_Pol, f) BC2, g) BC3 and BC4, h) BC5_Pol, i) GM1_Ant, j) GM2_Ant, k) GM3, l) GM4_SAR92, m) GM5, n) GM6_SUP05, and o) AL1_Pel. Colored circles indicate taxonomic information for each scaffold obtained using phylogenetic marker genes of the scaffold: *Bacteroidetes* (red), *Alphaproteobacteria* (blue), *Gammaproteobacteria* (green), *Betaproteobacteria* (purple), unclassified *Proteobacteria* (orange), *Cyanobacteria* (yellow), *Firmicutes* (brown), and others (pink). Putative target bins are indicated using arrows. **Figure S5.** Correlation of gene expression between PK and DC (PK-mRNA-TPM and DC-mRNA-TPM) for selected bins. **Figure S6.** COG-based transcriptome analysis of 12 genomes using a scatter plot of mRNA-TPM. Each point represents the relative abundance of a transcript assigned to a COG category. (A) total COGs; (B) transporter-related COGs; (C) GH-related COGs; (D) DMSP-, GB-, and glyoxylate shunt-related COGs; and (E) proteorhodopsin-related COGs. **Figure S7.** Gene expression levels of representative glycoside hydrolase genes from GM4_SAR92. GH3, glycoside hydrolase 3; GH16, glycoside hydrolase 16; GH42, glycoside hydrolase 42. **Figure S8.** Metabolism of dimethylsulfoniopropionate (DMSP), gene expression levels, and gene clusters across the reconstructed genomes. a) Relative abundance of *dmdA* transcripts from each bin, b) organization of the clusters of genes involved in DMSP utilization, and c) pathway for the utilization of DMSP. 1, DMSP demethylase (*dmdA*); 2, *O*-acetylhomoserine aminocarboxypropyltransferase; 3, alpha/beta hydrolase fold protein; 4, 3-hydroxyacyl-CoA dehydrogenase (*dmdC*); 5, acyl-CoA synthetase (*dmdB*); MMPA, methylmercaptopropionate; MMPA-CoA, 3-methylmercaptopropionyl-CoA; MTA-CoA, methylthioacryloyl-CoA. **Figure S9.** Synteny of GM1_Ant, GM2_Ant, and fosmic clone Ant4D3. Colors are assigned based on the COG classification. (DOCX 8210 kb)

Additional file 4: **Table S2.** List of single marker genes from 12 bins and their phylogenetic positions determined using the NCBI NR database (Excel). (XLSX 33 kb)

Additional file 5: **Table S5.** Information for the 500 most abundant genes in 12 genomes based on metatranscriptome data (a, PK; b, DC; and c, SI). Genes related to ribosome and mitochondrial biogenesis classified by KEGG and hypothetical proteins are marked in gray. (Excel) (XLSX 109 kb)
