# Correction to: Genomic and metatranscriptomic analyses of carbon remineralization in an Antarctic polynya

**DOI:** 10.1186/s40168-019-0655-0

**Published:** 2019-03-11

**Authors:** So-Jeong Kim, Jong-Geol Kim, Sang-Hoon Lee, Soo-Je Park, Joo-Han Gwak, Man-Young Jung, Won-Hyung Chung, Eun-Jin Yang, Jisoo Park, Jinyoung Jung, Yoonsoo Hahn, Jang-Cheon Cho, Eugene L. Madsen, Francisco Rodriguez-Valera, Jung-Ho Hyun, Sung-Keun Rhee

**Affiliations:** 1 Geologic Environment Research Division, Korea Institute of Geoscience and Mineral Resources, Daejeon, 34132 Republic of Korea; 2 Department of Microbiology, Chungbuk National University, Cheongju, 28644 Republic of Korea; 3 Division of Polar Ocean Environment, Korea Polar Research Institute, Incheon, 21990 Republic of Korea; 4 Department of Biology, Jeju National University, Jeju, 63243 Republic of Korea; 5 Department of Microbial Ecology, University of Vienna, 1090 Vienna, Austria; 6 Research Group of Gut Microbiome, Korea Food Research Institute, Sungnam, 13539 Republic of Korea; 7 Department of Life Science, Chung-Ang University, Seoul, 06974 Republic of Korea; 8 Department of Biological Sciences, Inha University, Incheon, 22212 Republic of Korea; 9 Department of Microbiology, Cornell University, Ithaca, NY 14853-8101 USA; 10 Evolutionary Genomics Group, División de Microbiología, Universidad Miguel Hernández, Apartado 18, San Juan de Alicante, 03550 Alicante, Spain; 11 Department of Marine Science and Convergence Engineering, Hanyang University ERICA Campus, Ansan, 15588 Republic of Korea

**Keywords:** Carbon remineralization, Genomics, Metatranscriptomics, Polynya

**Correction to: Microbiome**

**https://doi.org/10.1186/s40168-019-0643-4**

Following publication of the original article [1], the authors identified wrong citations in the maintext.

Page 3, right column: Additional file 1: Table S1 → Table 1.

Page 4, right column: detail in Additional file 1: Table S1 → detail in Additional file 2: Supplementary results.

Page 5, right column: Table 2 (see Additional file 1: Table S1) → Table 2 (see Additional file 2: Supplementary results).

Page 12, left column: Additional file 1: Table S10 → Additional file 1: Table S11.

Page 12, left column: Additional file 1: Table S12 → Additional file 5: Table S12.

Description of additional files on Page 12 and 13:

Table S10 and Table S12→ Table S12 and Table S10. Respectively.

Additional file 5 Table S5 → Additional file 5: Table S12.

Kim et al. [19] → Kim et al. [21]

## References

[1] Kim SJ, Kim JG, Lee SH, Park SJ, Gwak JH (2019). Genomic and metatranscriptomic analyses of carbon remineralization in an Antarctic polynya. Microbiome.

