# Supplementary material for: Genomic and metatranscriptomic analyses of carbon remineralization in an Antarctic polynya
Source: Microbiome. 2019 Feb 20;7:29. doi: 10.1186/s40168-019-0643-4 (PMC6383258; doi:10.1186/s40168-019-0643-4)
Supplement: Supplementary file 1 — Table S1. Diversity and abundance of bacterial 16S rRNA gene sequences obtained by the pyrosequencing of PCR amplicons in this study. Taxa with frequencies of < 1% were omitted from all samples. N/D, not detected. Table S3. DNA reads mapped to AL1_Pel and three Pelagibacter genomes. Table S4. Abundance (phylum level) of 16S rRNA gene among DNA reads. tr, < 0.1%; N/D, not detected. Table S5. Raw read classification based on NT database using Centrifuge. Table S6. Summary of metagenome and metatranscriptome data. Table S7. Spearman correlation coefficients for genes in the PK-mRNA-TPM and DC-mRNA-TPM datasets. Table S8. Bloom phase-specific gene expression and average fold changes in expression in 12 genome bins. Table S9. Summary of genes encoding representative transporters (TBDT, ABC, and TRAP) and SusD from 12 genomes. Table S10. Comparison of selected genes and pathways in GM1_Ant, GM2_Ant, GM4_SAR92, GM6_SUP05, and Roseobacter clades. Table S12. Genes in the pathway for vitamin B12 biosynthesis in GM1_Ant, GM2_Ant, and GM4_SAR92. (DOCX 73 kb) [file 40168_2019_643_MOESM1_ESM.docx]

**Supplementary Tables**

**Genomic and metatranscriptomic analyses of carbon remineralization in an Antarctic polynya**

So-Jeong Kim, Jong-Geol Kim, Sang-Hoon Lee, Soo-Je Park, Joo-Han Gwak, Man-Young Jung, Won-Hyung Chung, Eun-Jin Yang, Jisoo Park, Jinyoung Jung, Yoonsoo Hahn, Jang-Cheon Cho, Eugene L. Madsen, Francisco Rodriguez-Valera, Jung-Ho Hyun and Sung-Keun Rhee

**Supplementary Table S1.** Diversity and abundance of bacterial 16S rRNA gene sequences obtained by the pyrosequencing of PCR amplicons in this study. Taxa with frequencies of < 1% were omitted from all samples. N/D; not detected.

|  | Peak-1 | Declining-1 | Declining-2 | Peak-2 | Sea ice |
| --- | --- | --- | --- | --- | --- |
| Raw reads | 8108 | 6135 | 5456 | 11467 | 7634 |
| OTU*^$^ | 166 | 265 | 330 | 115 | 211 |
| Chao1 estimated richness^$^ | 289 | 633 | 745 | 237.2 | 476 |
| Shannon’s index for diversity^$^ | 2.205 | 3.181 | 3.300 | 1.774 | 3.114 |
| Simpson’s index for diversity (Inverse)^$^ | 0.7829 | 0.9007 | 0.9063 | 0.6945 | 0.9063 |
| Good’s coverage (%)^$^ | 98.5 | 97.0 | 96.4 | 98.9 | 98.1 |
| Taxonomy |  |  |  |  |  |
| *Bacteroidetes* |  |  |  |  |  |
| *Flavobacteriales* |  |  |  |  |  |
| Unclassified *Flavobacteriales* | tr | 6.95 | 9.17 | 1.47 | tr |
| Unclassifided *Cryomorphaceae* | tr | tr | tr | tr | 1.32 |
| Unclassified *Flavobacteriaceae* | 0.82 | 8.50 | 11.80 | 0.93 | 2.24 |
| *Polaribacter* | 37.37 | 9.86 | 7.76 | 50.50 | 14.49 |
| *Psychroserpens* | N/D | 1.08 | N/D | tr | N/D |
| *Proteobacteria* |  |  |  |  |  |
| *Alphaproteobacteria* |  |  |  |  |  |
| Unclassified *Alphaproteobacteria* | 0.52 | tr | tr | tr | 1.46 |
| *Rhodobacterales* |  |  |  |  |  |
| Unclassified *Rhodobacteraceae* | tr | 6.27 | 5.36 | tr | 2.74 |
| *Loktanella* | tr | 2.23 | 2.45 | tr | tr |
| *Octadecabacter* | 1.93 | 6.09 | 5.73 | 0.61 | 6.01 |
| *Pseudoruegeria* | tr | N/D | N/D | N/D | 1.37 |
| *Rickettsiales* |  |  |  |  |  |
| SAR11 clade | 11.68 | 25.00 | 23.08 | 4.04 | 22.68 |
| *Gammaproteobacteria* |  |  |  |  |  |
| Unclassified *Gammaproteobacteria* | tr | 1.11 | 1.04 | tr | tr |
| *Alteromonadales* |  |  |  |  |  |
| Unclassifided *Alteromonadales* | N/D | 2.11 | 1.45 | N/D | tr |
| Unclassifided *Alteromonadaceae* | tr | 1.85 | 0.86 | N/D | 0.53 |
| SAR92 clade | 8.49 | 2.57 | 2.98 | 6.66 | 4.72 |
| *Oceanospirillales* |  |  |  |  |  |
| Unclassified *Oceanospirillaceae* | 28.72 | 20.66 | 20.38 | 30.36 | 11.47 |
| SUP05 clade | 2.97 | tr | tr | 0.52 | 13.09 |
| *Thiotrichales* |  |  |  |  |  |
| Unclassified *Piscirickettsiaceae* | 1.12 | 2.34 | 4.07 | 3.35 | 7.04 |
| *Deltaproteobacteria* |  |  |  |  |  |
| SAR324 clade | 0.71 | N/D | tr | tr | 1.09 |

*An OTU was defined as containing sequences with a similarity of at least 97%. tr; < 0.5% abundance.

^$^Alpha diversity metrics were calculated for an even sequencing depth (5456 reads subsampled per sample).

**Supplementary Table S2.** List of single marker genes from 12 bins and their phylogenetic positions determined using the NCBI NR database (Refer to Additional Excel File).

**Supplementary Table S3.** DNA reads mapped to AL1_Pel and three *Pelagibacter* genomes.

|  | **PK** | **DC** | **SI** |
| --- | --- | --- | --- |
| AL1_Pel | 4,518,392 | 6,376,429 | 29,356,686 |
| Known *Pelagibacter* genomes* | 1,756,367 | 2,471,753 | 11,473,960 |

**Pelagibacter* sp. HTCC7211, *Pelagibacter* sp. IMCC9063, and *Pelagibacter ubique* HTCC1062

**Supplementary Table S4.** Abundance (phylum level) of 16S rRNA gene among DNA reads. tr, < 0.1%; N/D, not detected.

|  | **PK** | **DC** | **SI** |
| --- | --- | --- | --- |
| *Actinobacteria* | 0.4 | 0.5 | 0.6 |
| *Bacteroidetes* | 53.1 | 33.8 | 48.7 |
| *Cyanobacteria* | 1.9 | 2.1 | 2.4 |
| *Firmicutes* | 0.2 | 0.3 | 0.4 |
| *Proteobacteria* |  |  |  |
| *Alphaproteobacteria* | 0.9 | 4.4 | 4.4 |
| *Betaproteobacteria* | 0.1 | 0.1 | 0.7 |
| *Gammaproteobacteria* | 10.4 | 23.6 | 11.0 |
| *unclassified_Proteobacteria* | 23.9 | 18.7 | 19.1 |
| *Planctomycetes* | 0.1 | N/D | N/D |
| *Spirochaetes* | tr | N/D | 0.1 |
| *Verrucomicrobia* | 0.1 | 0.1 | 0.2 |
| unclassified_*Bacteria* | 6.8 | 16.2 | 12.4 |
| *Crenarchaeota* | 0.5 | 0.1 | tr |
| *Euryarchaeota* | 0.5 | tr | tr |
| unclassified*_Archaea* | 1.0 | N/D | tr |

**Supplementary Table S5.** Raw read classification based on NT database using Centrifuge.

|  | **DNA (%)** | | | **mRNA (%)** | | |
| --- | --- | --- | --- | --- | --- | --- |
|  | **PK** | **DC** | **SI** | **PK** | **DC** | **SI** |
| Bacteria | 21.7 | 13.2 | 22.8 | 36.7 | 12.9 | 24.5 |
| -*Bacteroidetes* | 7.6 | 3.0 | 2.0 | 17.6 | 3.1 | 6.5 |
| -*Alphaproteobacteria* | 0.8 | 1.7 | 6.7 | 0.7 | 1.7 | 4.0 |
| -*Gammaproteobacteria* | 1.8 | 2.3 | 3.0 | 6.3 | 4.2 | 6.7 |
| Archaea | 0.2 | 0.1 | 0.2 | 0.1 | 8.5 | 0.1 |
| Environmental samples | 0.1 | 0.1 | 0.3 | 0.2 | 0.3 | 0.5 |
| Eukaryota | 8.1 | 9.6 | 9.8 | 13.3 | 19.3 | 12.2 |
| Viruses | 0.4 | 0.2 | 0.3 | 0.2 | 0.1 | 0.1 |
| No hit | 69.6 | 76.9 | 66.7 | 49.5 | 58.8 | 62.5 |

**Supplementary Table S6.** Summary of metagenome and metatranscriptome data

|  | DNA | | | mRNA | | | Number of scaffolds | Sum of scaffolds (bp) |
| --- | --- | --- | --- | --- | --- | --- | --- | --- |
|  | PK | DC | SI | PK | DC | SI |  |  |
| Total reads | 337,950,538 | 212,798,116 | 170,986,196 | 50,843,842 | 59,884,236 | 56,859,202 |  |  |
| Total reads after removing Eukaroyote and virus reads^$^ | 309,206,576 | 191,844,778 | 153,752,466 | 43,701,612 | 47,909,398 | 49,553,468 |  |  |
| Reads mapped to binned scaffold | 95,625,154 | 31,124,872 | 30,823,615 | 18,012,957 | 3,553,189 | 6,570,980 | 3906 | 39,125,484 |
| BC1_Pol | 52,390,318 | 3,009,658 | 1,030,581 | 10,974,160 | 177,160 | 656,003 | 154 | 3,073,674 |
| BC2 | 1,014,742 | 2,570,072 | 102,048 | 135,381 | 108,888 | 83,525 | 297 | 2,131,182 |
| BC3 | 141,665 | 4,700,245 | 555,874 | 16,555 | 2,366 | 236,069 | 13 | 2,850,027 |
| BC4 | 95,244 | 2,482,625 | 489,488 | 12,007 | 15,652 | 390,404 | 29 | 1,803,780 |
| BC5_Pol | 7,446,937 | 956,834 | 191,457 | 1,143,691 | 48,582 | 97,287 | 280 | 1,958,181 |
| GM1_Ant | 17,868,419 | 3,318,956 | 2,871,983 | 2,808,861 | 532,718 | 1,092,578 | 36 | 1,997,708 |
| GM2_Ant | 2,615,640 | 3,993,729 | 1,815,473 | 511,028 | 325,912 | 706,401 | 68 | 2,190,547 |
| GM3 | 33,580 | 3,315,969 | 290,388 | 10,621 | 6,916 | 117,184 | 200 | 2,726,251 |
| GM4_SAR92 | 8,223,066 | 3,094,832 | 3,283,740 | 1,494,836 | 717,494 | 1,047,613 | 490 | 3,311,676 |
| GM5 | 2,156,799 | 42,466 | 371,475 | 646,481 | 119,319 | 95,714 | 127 | 1,399,375 |
| GM6_SUP05 | 1,096,572 | 45,371 | 3,121,133 | 101,005 | 980,103 | 360,151 | 281 | 2,906,510 |
| AL1_Pel | 2,542,172 | 3,594,115 | 16,699,975 | 158,331 | 518,079 | 1,688,051 | 1931 | 10,879,719 |
| Reads mapped to un-binned scaffold* | 76,427,515 | 50,482,954 | 86,490,648 | 9,304,687 | 8,220,363 | 22,598,370 | 32530 | 339,874,259 |
| /Reads mapped to un-binned bacterial scaffold |  |  |  |  |  |  |  |  |
| - Actinobacteria scaffolds | 77,258 | 147,766 | 363,709 | 10,108 | 11,852 | 58,868 | 219 | 2,518,644 |
| - Bacteroidetes scaffolds | 13,838,801 | 9,135,289 | 14,246,508 | 2,296,202 | 2,055,229 | 7,512,761 | 4253 | 68,142,923 |
| - Alphaproteobacteria scaffolds | 7,718,862 | 11,685,767 | 27,373,153 | 729,075 | 1,469,451 | 4,983,289 | 16927 | 142,707,927 |
| - Betaproteobacteria scaffolds | 1,084,852 | 351,233 | 1,954,142 | 89,604 | 172,787 | 398,050 | 1901 | 15,988,124 |
| - Gammaproteobacteria scaffolds | 17,963,943 | 15,901,617 | 15,725,757 | 3,574,489 | 2,316,362 | 5,704,079 | 6387 | 92,778,323 |
| - Delta/Epsilonproteobacteria scaffolds | 74,218 | 92,937 | 117,987 | 5,114 | 3,352 | 15,833 | 399 | 1,570,131 |
| - Unclassified Proteobacteria scaffolds | 16,961,893 | 1,717,512 | 4,174,737 | 948,461 | 257,851 | 400,838 | 1036 | 10,429,633 |
| - Unclassified Bacteria scaffolds | 3,486,295 | 1,651,667 | 5,298,906 | 194,995 | 176,903 | 661,456 | 1602 | 23,883,795 |
| /Reads mapped to un-binned archaeal scaffold |  |  |  |  |  |  |  |  |
| - Crenarchaeota | 546 | 1,074 | 6,081 | 3 | 35 | 214 | 21 | 91,452 |
| - Euryarchaeota | 23,471 | 20,049 | 281,441 | 312 | 2,537 | 14,410 | 158 | 2,676,693 |
| - Thaumarchaeota | 1054 | 993 | 4,553 | 25 | 133 | 257 | 25 | 59,266 |
| Reads mapped to Uniref90** | 17,167,012 | 21,092,244 | 5,057,470 | 1,627,152 | 8,414,430 | 2,107,645 |  |  |
| Unmapped reads | 165,897,869 | 131,190,290 | 53,671,933 | 14,756,816 | 27,721,416 | 18,276,473 |  |  |

^$^Based on Centrifuge

*Un-binned scaffold of SI metagenome

**Diamond blastx against Uniref90 with unmapped read to SI metagenome

**Supplementary Table S7.** Spearman correlation coefficients for genes in the PK-mRNA-TPM and DC-mRNA-TPM datasets.

| Bin | Correlation coefficient  (PK-mRNA-TPM: DC-mRNA-TPM) |
| --- | --- |
| BC1_Pol | 0.74 |
| BC2 | 0.82 |
| BC3 | 0.44 |
| BC4 | 0.58 |
| BC5_Pol | 0.72 |
| GM1_Ant | 0.90 |
| GM2_Ant | 0.87 |
| GM3 | 0.46 |
| GM4_SAR92 | 0.90 |
| GM5 | 0.74 |
| GM6_SUP05 | 0.87 |
| AL1_Pel | 0.68 |
| 12 Selected Bins | 0.61 |

Correlation coefficients of >0.05 were considered high (marked in bold), those between 0.5 and 0.3 were medium, those between 0.3 and 0.15 were low, those between 0.15 and −0.15 indicated no correlation, and those below −0.15 were negative. [1]

**Supplementary Table S8.** Bloom phase-specific gene expression and average fold changes in expression in 12 genome bins.

| **Bin** | **Total gene** | **PK-specific** | **DC-specific** | **No expression** | **Average fold change***  **(PK vs. DC)** |
| --- | --- | --- | --- | --- | --- |
| **BC1_Pol** | 2687 | 466 | 3 | 60 | 0.64 |
| **BC2** | 1669 | 65 | 17 | 86 | 0.36 |
| **BC3** | 2345 | 1033 | 53 | 759 | 0.48 |
| **BC4** | 1599 | 295 | 139 | 262 | 0.48 |
| **BC5_Pol** | 1368 | 216 | 2 | 92 | 0.68 |
| **GM1_Ant** | 1795 | 25 | 8 | 52 | 0.33 |
| **GM2_Ant** | 2023 | 49 | 26 | 76 | 0.32 |
| **GM3** | 3124 | 712 | 344 | 1172 | 0.45 |
| **GM4_SAR92** | 2489 | 42 | 31 | 150 | 0.39 |
| **GM5** | 1285 | 114 | 5 | 5 | 0.34 |
| **GM6_SUP05** | 1900 | 1 | 174 | 91 | 0.34 |
| **AL1_Pel** | 7287 | 63 | 2301 | 918 | 0.43 |

*Deseq2 was used for calculation.

**Supplementary Table S9.** Summary of genes encoding representative transporters (TBDT, ABC, and TRAP) and SusD from 12 genomes.

|  | **BC1_**  **Pol** | **BC5_**  **Pol** | **BC2** | **BC3** | **BC4** | **GM4_**  **SAR92** | **GM1_**  **Ant** | **GM2_**  **Ant** | **GM3** | **GM5** | **GM6_**  **SUP05** | **AL1_**  **Pel** |
| --- | --- | --- | --- | --- | --- | --- | --- | --- | --- | --- | --- | --- |
| **TBDT** |  |  |  |  |  |  |  |  |  |  |  |  |
| Cluster 3090 | 2 | 1 |  | 2 | 3 | 1 |  |  |  |  |  |  |
| Cluster 720 | 16 | 15 | 8 | 4 | 5 |  |  |  |  |  |  |  |
| Cluster 427 | 2 | 2 | 1 |  |  | 5 |  |  |  |  |  |  |
| Cluster 3303 | 1 | 1 | 1 | 1 | 1 | 1 |  |  |  |  |  |  |
| Cluster 410 | 1 | 1 | 1 | 2 | 1 | 14 |  |  | 1 |  |  |  |
| Cluster 973 | 1 | 3 | 7 | 5 | 5 | 1 |  |  | 1 |  | 1 |  |
| Cluster 180 | 7 | 6 | 4 | 3 | 1 |  |  |  |  |  |  |  |
| Cluster 1609 | 1 |  |  |  |  |  |  |  |  |  |  |  |
| Cluster 1856 | 4 | 1 |  | 1 |  | 2 |  |  |  |  |  |  |
| Cluster 767 | 1 |  |  | 1 |  |  |  |  |  |  |  |  |
| Unclassified TBDT | 5 | 2 | 6 | 5 | 3 | 1 | 1 |  | 3 | 6 | 1 |  |
| **SusD** |  |  |  |  |  |  |  |  |  |  |  |  |
| SusD | 10 | 5 | 3 | 1 | 2 |  |  |  |  |  |  |  |
| **ABC** |  |  |  |  |  |  |  |  |  |  |  |  |
| Amino acid | 2 | 2 | 1 | 3 |  |  | 12 | 12 | 11 | 1 | 6 | 29 |
| Branched amino acid |  |  |  |  |  |  | 14 | 7 |  |  | 3 | 36 |
| Dipeptide |  |  | 1 |  |  | 1 | 3 |  | 1 |  | 1 |  |
| Oligopeptide |  |  | 1 |  | 1 |  | 3 |  | 2 | 1 | 1 |  |
| Peptide |  |  | 2 |  | 2 | 4 | 8 |  | 5 | 1 | 14 | 5 |
| Phenylpropanoid |  |  |  |  |  |  |  | 1 |  |  |  | 4 |
| Monosaccharide | 1 | 1 |  |  |  |  | 11 | 8 | 16 |  | 31 | 47 |
| Disaccharide |  |  |  |  |  |  | 3 | 5 | 1 |  | 13 | 9 |
| Oligosaccharide |  |  |  | 1 |  |  |  | 3 | 6 |  | 5 | 1 |
| Glycoside |  |  |  |  |  |  | 1 | 3 | 1 |  |  | 4 |
| Sugar alcohol |  |  |  |  | 1 |  | 2 | 3 | 5 |  | 3 | 1 |
| Glycine betaine/Proline/Proline betaine |  |  |  |  | 1 |  | 3 | 9 | 4 |  | 18 | 25 |
| Glycerol/Glycerol-phosphate |  |  |  |  |  |  |  | 4 | 2 |  | 1 | 3 |
| Taurine |  |  |  |  |  |  | 6 |  | 2 |  | 1 | 16 |
| Opine/Polyamine |  |  |  |  | 1 | 4 | 2 | 6 |  |  | 14 | 2 |
| Steroid/lipid | 2 |  | 1 | 1 | 2 |  |  |  |  |  |  |  |
| Cyanate/Nitrite/Nitrate |  |  |  |  |  |  | 2 | 3 |  |  |  |  |
| Urea |  |  |  |  |  |  |  | 1 | 5 |  |  | 5 |
| Mo/W |  |  |  |  |  | 4 | 2 | 4 | 1 | 3 | 5 | 1 |
| Fe/Zn/Cu/Mn | 6 | 4 | 1 | 6 | 6 | 9 | 3 | 3 | 7 | 1 | 6 | 14 |
| Ferric iron-siderophore | 2 | 1 | 1 | 1 | 2 | 2 |  | 1 | 2 | 1 | 2 | 3 |
| Ferrichrome/Heme | 2 |  |  | 1 | 1 | 8 |  |  | 4 |  |  |  |
| Ni/Co | 2 |  |  |  |  |  |  | 2 |  |  | 1 | 2 |
| Vitamin B complex |  |  |  | 1 | 1 | 1 | 2 | 6 | 4 |  | 1 | 7 |
| Lipoprotein | 3 | 1 | 1 | 2 | 2 | 3 | 2 | 2 | 1 |  | 1 | 6 |
| Nucleoside |  |  |  |  |  |  | 3 | 6 | 6 |  | 8 | 2 |
| Phosphonate/Phosphate |  |  |  |  |  | 4 |  |  | 4 |  |  | 5 |
| Sugar phosphate |  | 1 |  |  |  |  | 1 | 2 | 1 |  | 5 | 2 |
| Phthalate/Protocatechuate |  |  |  |  |  |  | 1 | 1 | 2 |  |  | 2 |
| Sulfate/Thiosulfate |  |  |  |  |  | 1 | 3 | 2 |  |  | 2 |  |
| Unclassified ABC | 3 | 1 | 1 | 2 | 1 |  | 1 | 2 | 4 |  | 3 | 2 |
| **TRAP** |  |  |  |  |  |  |  |  |  |  |  |  |
| C4-dicarboxylate |  |  |  |  |  |  | 1 | 7 | 9 |  |  | 25 |
| Mannitol/chloroaromatic |  |  |  |  |  | 3 | 1 | 3 | 1 |  | 3 | 23 |
| Unclassified TRAP |  |  |  |  |  |  | 7 | 6 | 2 |  | 2 | 32 |

**Supplementary Table S10.** Genes in the pathway for vitamin B_12_ biosynthesis in GM1_Ant, GM2_Ant, and GM4_SAR92.

| **Genes** | **GM1_Ant** | **GM2_Ant** | **GM4_**  **SAR92** |
| --- | --- | --- | --- |
| CbiB/CobD COG1270 Cobalamin biosynthesis protein | + | + | + |
| CobQ COG1492 Cobyric acid synthase | + | - | + |
| CobN COG1429 Cobalamin biosynthesis protein | + | + | - |
| CobM COG2875 Precorrin-4 methylase | + | + | - |
| CobJ COG1010 Precorrin-3B methylase | + | + | - |
| CobF COG2243 Precorrin-2 methylase | + | + | - |
| CobH COG2082 Precorrin isomerase | + | + | - |
| CobK COG2099 Precorrin-6x reductase | + | + | - |
| CbiD COG1903 Cobalamin biosynthesis protein | + | + | - |
| CobB COG1797 Cobyrinic acid a,c-diamide synthase | + | + | - |
| CobS COG0368 Cobalamin-5-phosphate synthase | + | + | + |
| CbiX Cobalamin (vitamin B12) biosynthesis CbiX domain containing protein | + | + | - |
| CobU COG2087 Adenosyl cobinamide kinase | + | + | + |
| CobC cobalamin biosynthetic protein | + | + | + |

**Supplementary Table S11.** Comparison of selected genes and pathways in GM1_Ant, GM2_Ant, GM4_SAR92, GM6_SUP05, and *Roseobacter* clades.

|  | **GM1_Ant** | **GM2_Ant** | **GM4_SAR92** | **GM6_SUP05** | ***Planktomarina temperata**** | ***Octadecabacter arcticus**** | ***Octadecabacter antarcticus**** | ***Sulfitobacter* sp. EE-36*** | ***Sulfitobacter* sp. NAS14.1*** |
| --- | --- | --- | --- | --- | --- | --- | --- | --- | --- |
| **Trophic strategy** |  |  |  |  |  |  |  |  |  |
| Photosynthesis cluster | **-** | **-** | **-** | **-** | **+** | **-** | **-** | **-** | **-** |
| Proteorhodopsin | **+** | **+** | **+** | **+** | **-** | **-** | **-** | **-** | **-** |
| Xanthothodopsin | **-** | **-** | **-** | **-** | **-** | **+** | **+** | **-** | **-** |
| **Aromatic degradation** |  |  |  |  |  |  |  |  |  |
| B-ketoadipate | **-** | **-** | **-** | **-** | **+** | **-** | **-** | **+** | **+** |
| gentisate pathway | **-** | **-** | **-** | **-** | **-** | **-** | **-** | **-** | **-** |
| benzoate | **-** | **-** | **-** | **-** | **+** | **-** | **-** | **-** | **-** |
| phenylacetic acid | **+** | **+** | **+** | **+** | **+** | **-** | **-** | **+** | **+** |
| homoprotocatechuate | **-** | **-** | **-** | **-** | **+** | **-** | **-** | **-** | **-** |
| homogentisate pathway | **-** | **-** | **-** | **-** | **-** | **-** | **-** | **+** | **+** |
| **Carbon monoxide utilization** |  |  |  |  |  |  |  |  |  |
| group I CO DH | **-** | **-** | **-** | **-** | **+** | **+** | **+** | **-** | **-** |
| group II CO DH | **-** | **-** | **-** | **-** | **+** | **+** | **+** | **+** | **+** |
| **C1 compound utilization** |  |  |  |  |  |  |  |  |  |
| C1 incorp (serine) | - | - | - | **-** | **+** | **-** | **-** | **-** | **-** |
| MeOH oxidation | -? | -? | - | **-** | **-** | **-** | **-** | **-** | **-** |
| TMA oxidation | - | - | - | **-** | **-** | **+** | **+** | **-** | **-** |
| formaldehyde oxidation | **+** | **+** | **+** | **-** | **+** | **+** | **+** | **+** | **+** |
| formate oxidation | **+** | **+** | **+** | **+** | **+** | **+** | **-** | **+** | **+** |
| **C2 compound utilization** |  |  |  |  |  |  |  |  |  |
| ethylmalonyl pathway | **-** | **-** | **-** | **-** | **+** | **+** | **+** | **+** | **+** |
| glyoxylate shunt | **+** | **+** | **+** | **+** | **-** | **-** | **-** | **-** | **-** |
| **Motility, sensing, and attachment** |  |  |  |  |  |  |  |  |  |
| chemotactic ability | **-** | **+** | **-** | **-** | **+** | **-** | **-** | **-** | **-** |
| flp pilus (type IV) | +? | - | + | **-** | **-** | **-** | **+** | **+** | **+** |
| **Sulfur related** |  |  |  |  |  |  |  |  |  |
| DMSP demethylase (dmdA) | + | + | + | **+** | **+** | **+** | **+** | **-** | **-** |
| sulfur oxidation (SOX) | - | + | + | **+** | **+** | **-** | **-** | **+** | **+** |
| **vitamin related** |  |  |  |  |  |  |  |  |  |
| biotin synthesis | +? | +? | - | **+** | **-** | **-** | **-** | **-** | **-** |
| cobalamin synthesis | + | + | +? | **-** | **+** | **+** | **+** | **+** | **+** |
| thiamine synthesis | - | - | + | **-** | **-** | **+** | **+** | **+** | **+** |
| **Nitrogen related** |  |  |  |  |  |  |  |  |  |
| nitrate reduction (dis) | **-** | **-** | **-** | **-** | **-** | **-** | **-** | **-** | **-** |
| nitrate reduction (ass) | **-** | **-** | **-** | **-** | **-** | **-** | **+** | **+** | **+** |
| nitrite reduction (dis) | **-** | **-** | **-** | **-** | **-** | **-** | **+** | **-** | **-** |
| nitrite reduction (ass) | **-** | **-** | **-** | **-** | **-** | **-** | **+** | **+** | **+** |
| urease | **-** | **-** | **-** | **-** | **+** | **+** | **+** | **+** | **+** |

*Data from Voget et al. [2] and Newton et al. [3]

? indicates that a gene cluster was not complete.

**Supplementary Table S12.** Information for the 500 most abundant genes in 12 genomes based on metatranscriptome data (a, PK; b, DC; and c, SI). Genes related to ribosome and mitochondrial biogenesis classified by KEGG and hypothetical proteins are marked in grey. (Refer to Additional Excel File)

**References**

1. Myhre S, Lingjaerde OC, Hennessy BT, Aure MR, Carey MS, Alsner J, Tramm T, Overgaard J, Mills GB, Borresen-Dale AL *et al*: **Influence of DNA copy number and mRNA levels on the expression of breast cancer related proteins**. *Mol Oncol* 2013, **7**(3):704-718.

2. Voget S, Wemheuer B, Brinkhoff T, Vollmers J, Dietrich S, Giebel HA, Beardsley C, Sardemann C, Bakenhus I, Billerbeck S *et al*: **Adaptation of an abundant *Roseobacter* RCA organism to pelagic systems revealed by genomic and transcriptomic analyses**. *ISME J* 2015, **9**(2):371-384.

3. Newton RJ, Griffin LE, Bowles KM, Meile C, Gifford S, Givens CE, Howard EC, King E, Oakley CA, Reisch CR *et al*: **Genome characteristics of a generalist marine bacterial lineage**. *ISME J* 2010, **4**(6):784-798.
