# Supplementary material for: Genomic and metatranscriptomic analyses of carbon remineralization in an Antarctic polynya
Source: Microbiome. 2019 Feb 20;7:29. doi: 10.1186/s40168-019-0643-4 (PMC6383258; doi:10.1186/s40168-019-0643-4)

**Supplementary Results and Methods**

**Genomic and metatranscriptomic analyses of carbon remineralization in an Antarctic polynya**

So-Jeong Kim, Jong-Geol Kim, Sang-Hoon Lee, Soo-Je Park, Joo-Han Gwak, Man-Young Jung, Won-Hyung Chung, Eun-Jin Yang, Jisoo Park, Jinyoung Jung, Yoonsoo Hahn, Jang-Cheon Cho, Eugene L. Madsen, Francisco Rodriguez-Valera, Jung-Ho Hyun and Sung-Keun Rhee

**Supplementary Methods**

***Phytoplankton composition and biomass analysis***

To perform the phytoplankton composition analysis, water samples were obtained from the Amundsen Sea using a Niskin rosette sampler. Each water sample (300 mL) was preserved with glutaraldehyde (1 % final concentration) and stored at 4 °C before staining [proflavin (0.33 %) and 40-6-diamidino-2-phenylindole (DAPI; 50 μg mL-1 final concentration)] and filtration (0.8 and 8 μm Nuclepore filters) to determine the abundance of autotrophic picoflagellates (APF), nanoflagellates (ANF) and dinoflagellates (ADF). Autotrophic organisms were distinguished from heterotrophs by the presence of chlorophyll, which was visualized as red fluorescence under blue light illumination. Most of *P. antarctica* cells were present in solitary form in the water column. Solitary flagellated cells were distinguished from other autotrophic flagellates based on cell size (about 3–6 μm). For diatoms, seawater samples were preserved with acid Lugol’s iodine solution and then stored in the dark. To estimate the carbon biomass of phytoplankton, the cell volume was calculated by measuring cell dimensions with an ocular micrometer in the microscope [1]. The conversion factors and equations were used to transform cell volumes into carbon biomass as follows: 220 fg C μm^-3^ for APF [2]; carbon (pg) = 0.216 × (volume, um^3^)^0.939^ for ANF and ADF [3]; carbon (pg) = 0.288 × (volume, μm^3^)^0.811^ for diatom [3]; and 3.33 pg C μm^-3^ for *P. antarctica* [4].

**Supplementary Results**

***Distribution of dominant OTUs***

The major OTU assigned to *Polaribacter,* which was closely related to uncultured *Polaribacter* sp. Vis St3 (Supplementary Fig. S2a), and the major OTU assigned to unclassified *Oceanospillaceae,* which was related to uncultivated Ant4D3 (Supplementary Fig. S2b), were abundant in each clade during all three phases of the bloom.

The Ant4D3 16S rRNA gene was from the fosmid clones from Antarctic coastal water belonging to *Gammaproteobacteria* [5], an abundant bacteria in both polar oceans [6]. The SAR92 clade was quite abundant during the peak bloom. In the southern North Sea, this clade was highly abundant and active in the phytoplankton bloom [7]. During bloom decline, unclassified *Flavobacteriales* and unclassified *Flavobacteriaceae* increased. *Flavobacteria* was particularly abundant during high primary production [8]. Additionally, *Flavobacteria* is associated with the decline phase of phytoplankton blooms because it is specialized for attachment and growth on detrital particles according to a metaproteomic study [9].

***Phylogenetic analysis of the 12 reconstructed genomes***

BC1 and BC5 were specifically affiliated with the genus *Polaribacter* (Appendix I) with an ANI of 91.8% and were designated BC1_Pol and BC5_Pol, respectively. GM1, GM2, GM3, GM4, GM5, and GM6 belonged to *Gammaproteobacteria* (Appendix I). GM1 and GM2 were assigned to the ‘Ant4D3’ clade following a fosmid clone of an unclassified *Oceanospirillaceae* [5] (designated GM1_Ant and GM2_Ant, respectively). The phylogenetic tree showed that GM1_Ant and GM2_Ant were closely related, with an ANI of 78.2%. Synteny of the genes of GM1_Ant and the fosmid clone Ant4D3 was highly conserved, with a high average nucleotide similarity (average 99.1%, Supplementary Fig. S9). AL1 contained a partial 16S rRNA gene (542 bp), which exhibited a similarity of 98% with that of *Pelagibacter ubique* HTCC1062 belonging to the SAR11 clade of *Alphaproteobacteria* (designated AL1_Pel). AL1_Pel included several unresolved bins of the SAR11 clade, which might be inseparable, as shown in Supplementary Fig. S4d and supported by the large total scaffold size (11.3 Mb) and contamination (98.9%, see Table 2).

***Utilization of glycine betaine (GB) and others***

Metabolic pathways for GB utilization were widely observed in the abundant *Gammaproteobacteria*, i.e., GM1_Ant, GM2_Ant, GM3, GM4_SAR92, and GM6_SUP05. Although putative genes for betaine-homocysteine methyltransferase were absent from all of the genomes, putative genes for GB:corrinoid methyltransferases (MtgB), recently identified as a GB methyltransferase [10], were present in all of the reconstructed genomes. The *MtgB* gene was colocalized with dimethylglycine dehydrogenase (Appendix II). Co-localization of putative genes encoding carnitine dehydrogenase and/or choline dehydrogenase with GB utilization indicates the potential use of various quaternary ammonium compounds for GM1_Ant and GM2_Ant. Transcripts from genes in this pathway, such as *mtgB* and *dmgdh,* were overrepresented in the GM1_Ant, GM2_Ant, and GM4_SAR92 bins in PK, DC, and SI, respectively (Supplementary Fig. S5b and c).

The potential utilization of taurine, glycerol, and mannitol by GM1_Ant, GM2_Ant, and AL1_Pel was suggested by the presence and expression of key genes involved in the uptake and degradation of these solutes (see above) in DC and SI. Our results suggest that compatible solutes are important substrates of GM1_Ant, GM2_Ant, and GM4_SAR92 in the polynya, especially in the decline phase of the bloom.

***Cobalamin synthesis***

Both GM1_Ant and GM2_Ant contained nearly complete sets of genes for vitamin B12 biosynthesis enzymes (Supplementary Table 12) [11]. Vitamin B12 is a limiting factor for the growth of *Phaeocystis* in the Antarctic Ocean [12, 13] and is required for key metabolic pathways, such as GB:corrinoid methyltransferases. Other studies have reported that *Oceanospirillaceae* plays an important role in cobalamin production in polynya [14, 15]. These results indicate that GM1_Ant and GM2_Ant are potential sources of vitamin B12 for the development and decay of phytoplankton blooms in the ASP.

**References**

1. Edler L: **Recommendations on methods for marine biological studies in the Baltic Sea. Phytoplankton and chlorophyll**. *Publication-Baltic Marine Biologists BMB (Sweden)* 1979.

2. Børsheim KY, Bratbak G: **Cell volume to cell carbon conversion factors for a bacterivorous *Monas* sp. enriched from seawater**. *Mar Ecol Prog Ser* 1987, **36**(17):ll.

3. Menden-Deuer S, Lessard EJ: **Carbon to volume relationships for dinoflagellates, diatoms, and other protist plankton**. *Limnol Oceanogr* 2000, **45**(3):569-579.

4. Mathot S, Smith WO, Carlson CA, Garrison DL, Gowing MM, Vickers CL: **Carbon partitioning within *Phaeocystis antarctica* (*Prymnesiophyceae*) colonies in the Ross Sea, Antarctica**. *J Phycol* 2000, **36**(6):1049-1056.

5. Grzymski JJ, Carter BJ, DeLong EF, Feldman RA, Ghadiri A, Murray AE: **Comparative genomics of DNA fragments from six Antarctic marine planktonic bacteria**. *Appl Environ Microbiol* 2006, **72**(2):1532-1541.

6. Straza TR, Ducklow HW, Murray AE, Kirchman DL: **Abundance and single‐cell activity of bacterial groups in Antarctic coastal waters**. *Limnol Oceanogr* 2010, **55**(6):2526-2536.

7. Wemheuer B, Güllert S, Billerbeck S, Giebel H-A, Voget S, Simon M, Daniel R: **Impact of a phytoplankton bloom on the diversity of the active bacterial community in the southern North Sea as revealed by metatranscriptomic approaches**. *FEMS Microbiol Ecol* 2014, **87**(2):378-389.

8. Abell GC, Bowman JP: **Ecological and biogeographic relationships of class *Flavobacteria* in the Southern Ocean**. *FEMS Microbiol Ecol* 2005, **51**(2):265-277.

9. Williams TJ, Long E, Evans F, Demaere MZ, Lauro FM, Raftery MJ, Ducklow H, Grzymski JJ, Murray AE, Cavicchioli R: **A metaproteomic assessment of winter and summer bacterioplankton from Antarctic Peninsula coastal surface waters**. *ISME J* 2012, **6**(10):1883-1900.

10. Ticak T, Kountz DJ, Girosky KE, Krzycki JA, Ferguson DJ, Jr.: **A nonpyrrolysine member of the widely distributed trimethylamine methyltransferase family is a glycine betaine methyltransferase**. *Proc Natl Acad Sci U S A* 2014, **111**(43):E4668-4676.

11. King GA: **Evolution of the coenzymes**. *Biosystems* 1980, **13**(1-2):23-45.

12. Bertrand EM, Saito MA, Jeon YJ, Neilan BA: **Vitamin B_12_ biosynthesis gene diversity in the Ross Sea: the identification of a new group of putative polar B_12_ biosynthesizers**. *Environ Microbiol* 2011, **13**(5):1285-1298.

13. Bertrand EM, Saito MA, Rose JM, Riesselman CR, Lohan MC, Noble AE, Lee PA, DiTullio GR: **Vitamin B_12_ and iron colimitation of phytoplankton growth in the Ross Sea**. *Limnol Oceanogr* 2007, **52**(3):1079-1093.

14. Delmont TO, Eren AM, Vineis JH, Post AF: **Genome reconstructions indicate the partitioning of ecological functions inside a phytoplankton bloom in the Amundsen Sea, Antarctica**. *Front Microbiol* 2015, **6**:1090.

15. Bertrand EM, McCrow JP, Moustafa A, Zheng H, McQuaid JB, Delmont TO, Post AF, Sipler RE, Spackeen JL, Xu K *et al*: **Phytoplankton-bacterial interactions mediate micronutrient colimitation at the coastal Antarctic sea ice edge**. *Proc Natl Acad Sci U S A* 2015, **112**(32):9938-9943.

**Appendix I.** Neighbor-joining trees based on partial amino acid sequences of *rpoB* gene obtained from genome bins and their nearest neighbors. Proteins of bins of a) *Bacteroidetes* and b) *Gammaproteobacteria* were shown.


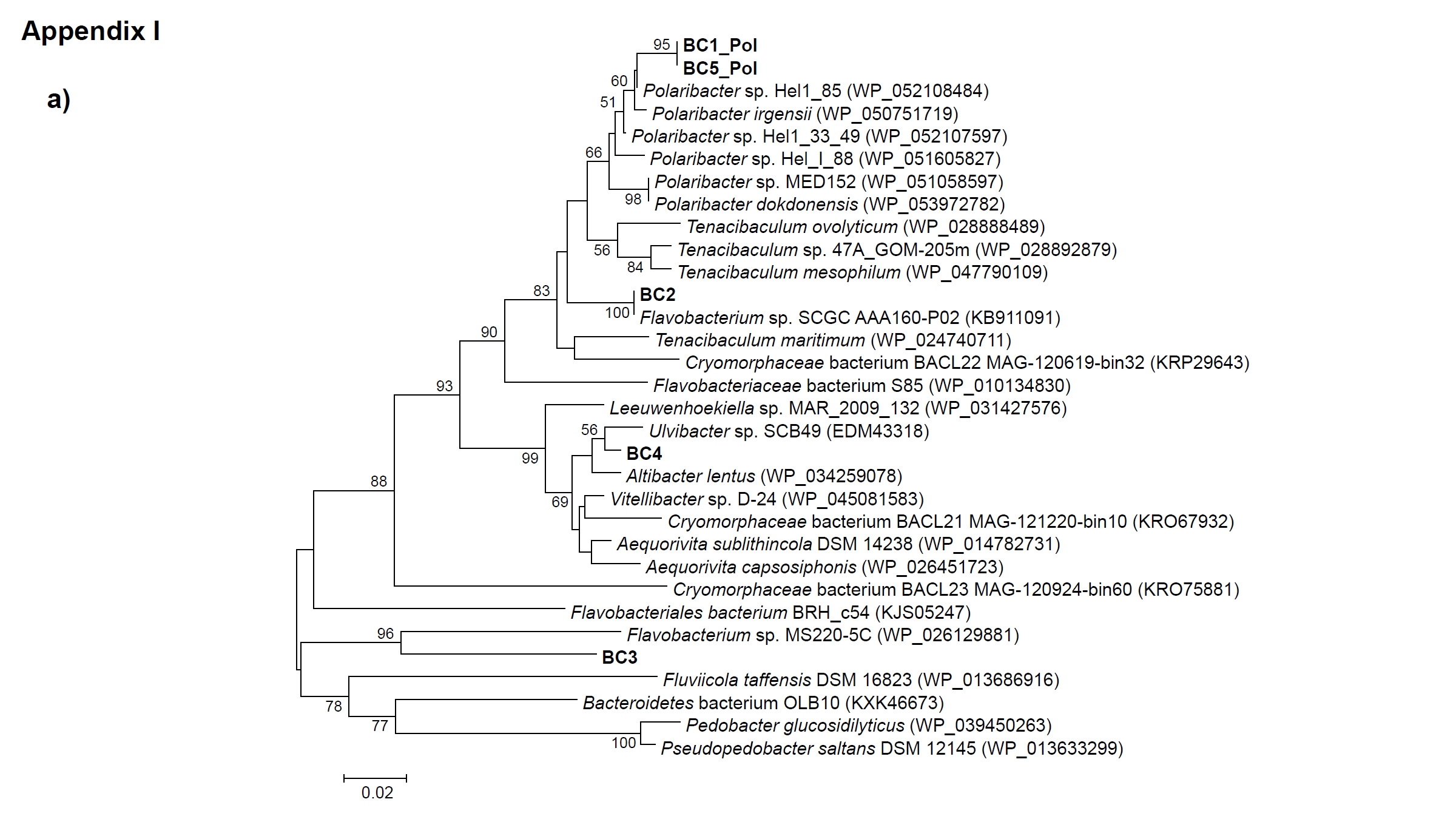


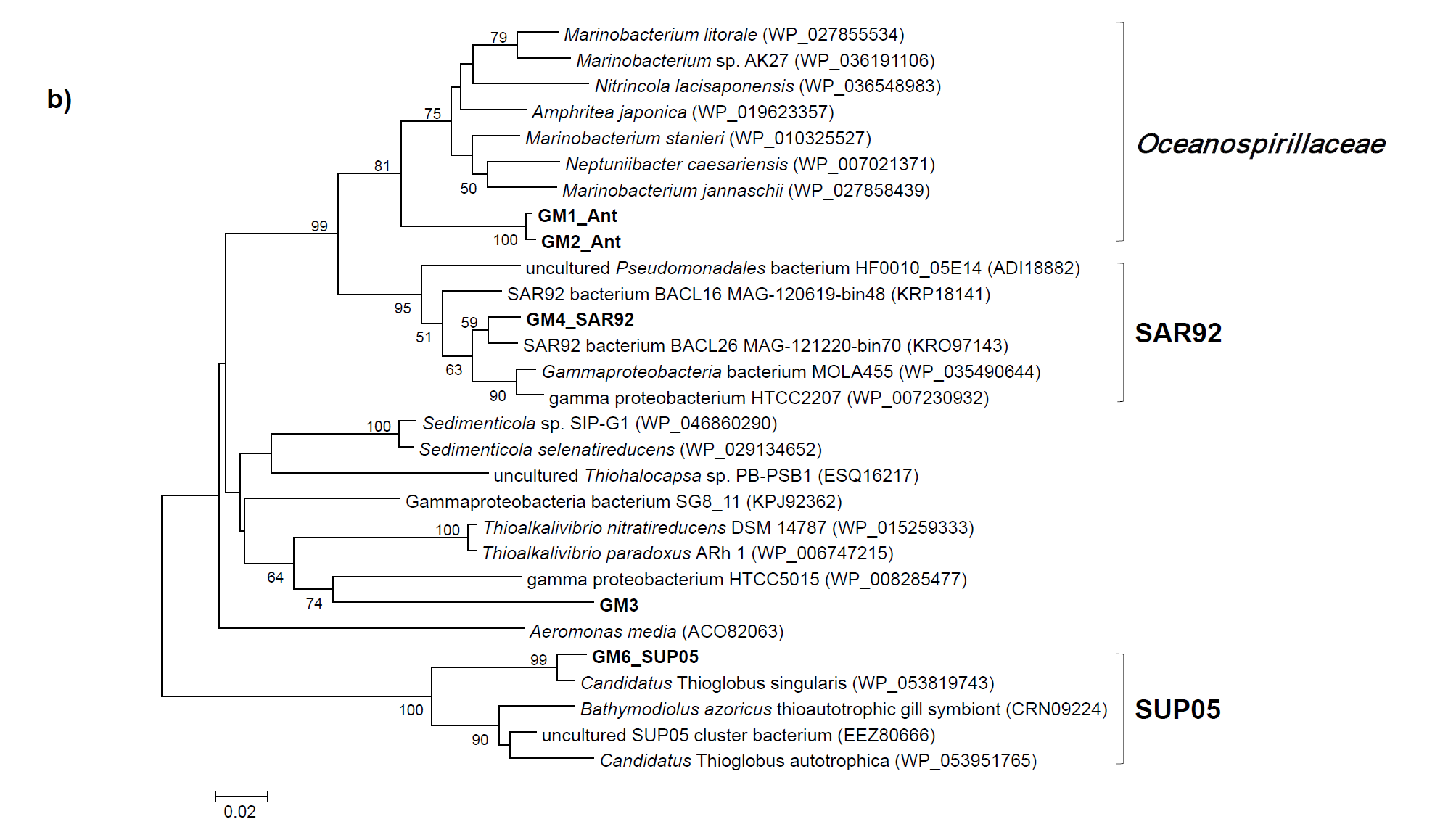


**Appendix II.** Metabolism of glycine betaine (GB), gene expression levels, and gene clusters across the reconstructed genomes. a) Organization of the clusters of genes involved in GB utilization. b) Pathway for the utilization of GB and the c) relative abundance of related genes for transcript reads from each bin.

1, Glycine betaine:corrinoid methyltransferase (mtgB); 2, Corrinoid protein; 3, Methionine synthase I, cobalamin-binding domain (metH); 4, Dimethylglycine dehydrogenase (dmgdh); 5, Sarcosine oxidase subunits (sox); 6, Choline dehydrogenase; 7, ABC transporter; 8, Carnitine dehydrogenase; 9,5,10-methylenetetrahydrofolate reductase (metF); 10, Formyltetrahydrofolate synthetase; 11, glycine betaine:H+ symporter (proP).


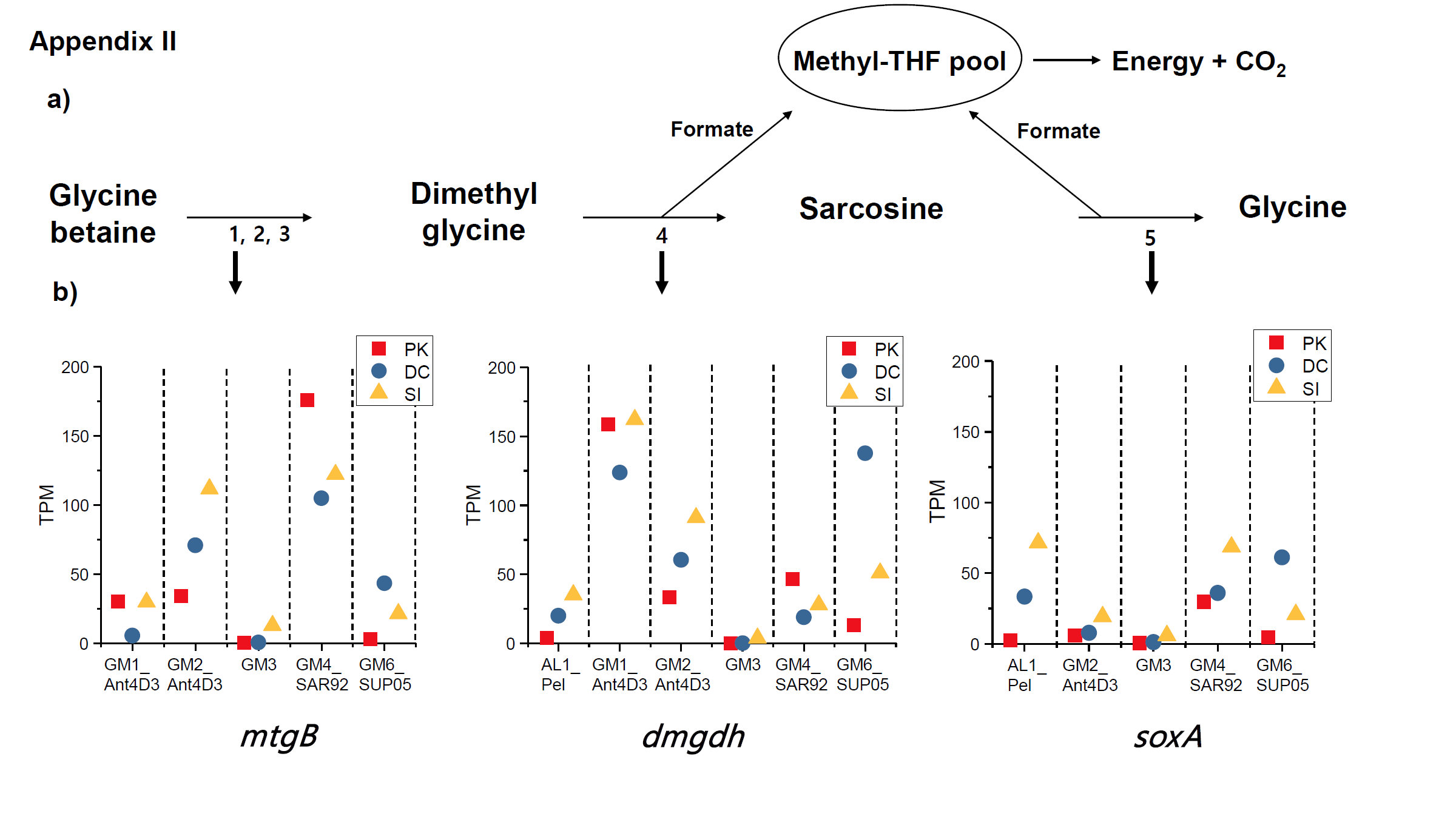


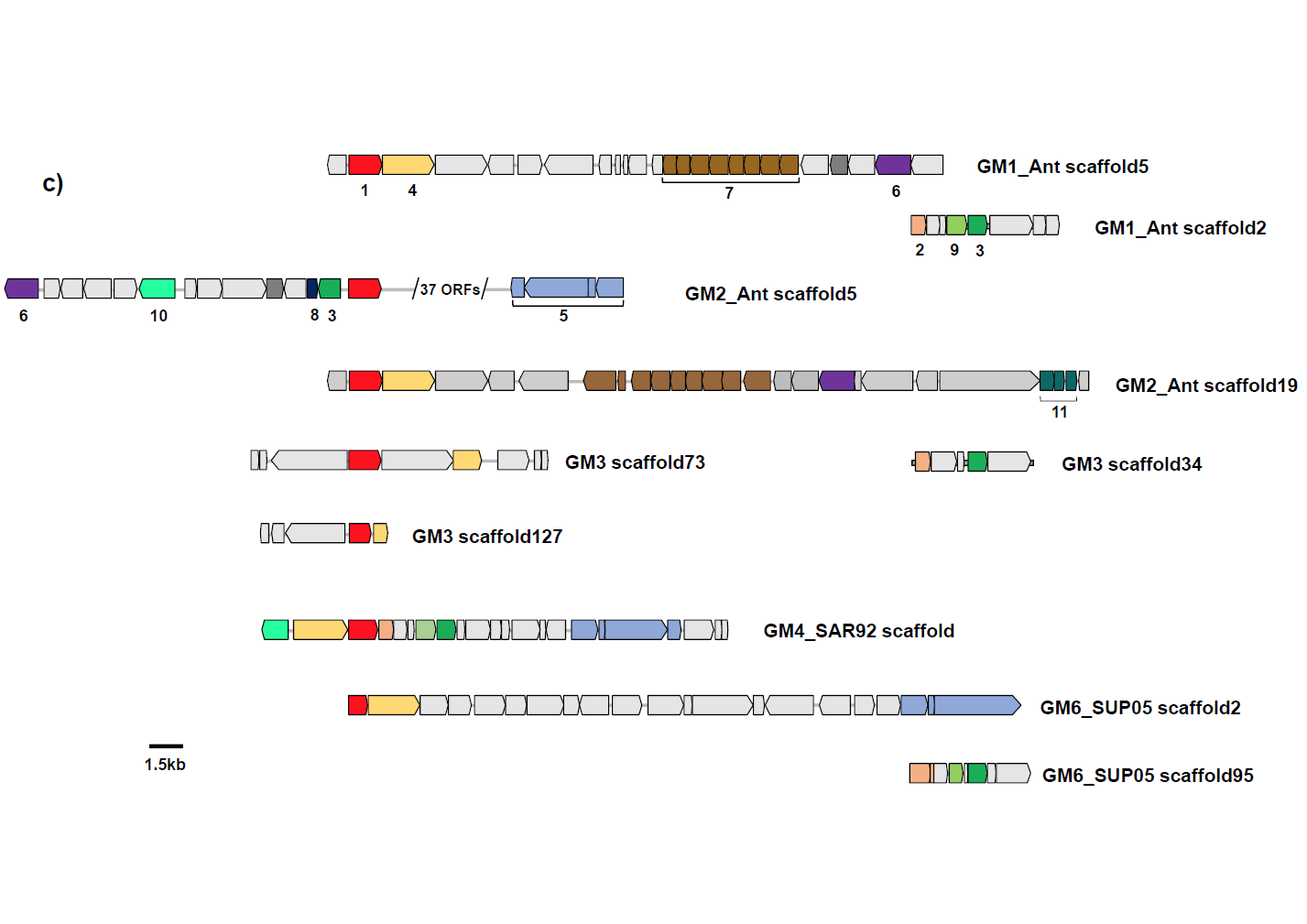

Supplement: Supplementary file 2 — Supplementary Results and Methods. (DOCX 939 kb) [file 40168_2019_643_MOESM2_ESM.docx]
