# Supplementary material for: Genomic and metatranscriptomic analyses of carbon remineralization in an Antarctic polynya
Source: Microbiome. 2019 Feb 20;7:29. doi: 10.1186/s40168-019-0643-4 (PMC6383258; doi:10.1186/s40168-019-0643-4)
Supplement: Supplementary file 3 — Figure S1. A) Satellite-based surface chlorophyll-a concentrations (mg/m3) and photosynthetically available radiance (PAR) in the polynya of the Amundsen Sea during the sampling cruises. B) Detailed satellite-based surface chlorophyll-a concentrations (mg/m3) and sampling day of this study. GPS position polynya center station, 73.25–73.75S, 114.25–113.75 W. The data constructed from multi ocean color sensors were obtained from the Globcolour webpage (http://hermes.acri.fr/). Figure S2. Neighbor-joining tree of the most abundant OTUs in a) Polaribacter, b) Oceanospirillaceae, c) SAR92, and d) SAR11 from PK, DC, and SI. The representative sequences of each OTU selected by QIIME are highlighted in bold and coded as follows: OTU N (=OTU number), site (PK, DC, or SI), and percentage of the total read number. Bootstrap values of ≥ 50% are shown. Figure S3. NMDS plot showing relationships between bacterial compositions and environmental variables. Bray–Curtis stress 0.117. *indicates p < 0.05 for the environmental parameter. Samples from station 1 to station 26 from Kim et al. [19] were reanalyzed. Samples from the polynya center in this study are represented as filled squares; green indicates the peak phase of the bloom, and red indicates the declining phase of the bloom. Filled triangles represent samples obtained under sea ice. Samples from the previous study are represented as empty squares for the polynya center (station 13), empty diamonds for the polynya margin (station 8), empty pentagons for the polynya ice shelf (station 11), empty circles for the open ocean (station 1), and empty triangles for water under sea ice (station 26). Figure S4. Differential coverage plot of scaffolds for DNA reads obtained from a) PK, b) DC, and c, d) SI. Samples used for coverage calculation are marked on the X-axis (decline phase of the bloom) and Y-axis (peak phase of the bloom). Summary of a principal component analysis of the tetranucleotide frequencies of scaffolds selected from [file 40168_2019_643_MOESM3_ESM.docx]

**Supplementary Figures**

**Genomic and metatranscriptomic analyses of carbon remineralization in an Antarctic polynya**

So-Jeong Kim, Jong-Geol Kim, Sang-Hoon Lee, Soo-Je Park, Joo-Han Gwak, Man-Young Jung, Won-Hyung Chung, Eun-Jin Yang, Jisoo Park, Jinyoung Jung, Yoonsoo Hahn, Jang-Cheon Cho, Eugene L. Madsen, Francisco Rodriguez-Valera, Jung-Ho Hyun and Sung-Keun Rhee

Supplementary Fig. S1. a) Satellite-based surface chlorophyll-a concentrations (mg/m^3^) and photosynthetically available radiance (PAR) in the polynya of the Amundsen Sea during the sampling cruises. b) Detailed satellite-based surface chlorophyll-a concentrations (mg/m^3^) and sampling day of this study. GPS position: polynya center station, 73.25–73.75S, 114.25–113.75W. The data constructed from multi ocean color sensors were obtained from the Globcolour webpage (http://hermes.acri.fr/).

Supplementary Fig. S2. Neighbor-joining tree of the most abundant OTUs in a) Polaribacter, b) Oceanospirillaceae, c) SAR92, and d) SAR11 from PK, DC, and SI. The representative sequences of each OTU selected by QIIME are highlighted in bold and coded as follows: OTU N (=OTU number), site (PK, DC, or SI), percentage of the total read number. Bootstrap values of ≥50% are shown.

Supplementary Fig. S3. NMDS plot showing relationships between bacterial compositions and environmental variables. Bray–Curtis, stress 0.117. *indicates p < 0.05 for the environmental parameter. Samples from Station 1 to Station 26 from Kim et al. [1] were re-analyzed. Samples from the polynya center in this study are represented as filled squares; green indicates the peak phase of the bloom and red indicates the declining phase of the bloom. Filled triangles represent samples obtained under sea ice. Samples from the previous study are represented as empty squares for the polynya center (station 13); empty diamonds for the polynya margin (station 8); empty pentagons for the polynya ice shelf (station 11); empty circles for the open ocean (station 1), and empty triangles for water under sea ice (station 26).

Supplementary Fig. S4. Differential coverage plot of scaffolds for DNA reads obtained from a) PK, b) DC, and c, d) SI. Samples used for coverage calculation are marked on the X-axis (decline phase of the bloom) and Y-axis (peak phase of the bloom). Summary of a principal component analysis of the tetranucleotide frequencies of scaffolds selected from differential coverage plot: e) BC1_Pol, f) BC2, g) BC3 and BC4, h) BC5_Pol, i) GM1_Ant, j) GM2_Ant, k) GM3, l) GM4_SAR92, m) GM5, n) GM6_SUP05, and o) AL1_Pel. Colored circles indicate taxonomic information for each scaffold obtained using phylogenetic marker genes of the scaffold; Bacteroidetes (red), Alphaproteobacteria (blue), Gammaproteobacteria (green), Betaproteobacteria (purple), unclassified Proteobacteria (orange), Cyanobacteria (yellow), Firmicutes (brown), and others (pink). Putative target bins are indicated using arrows.


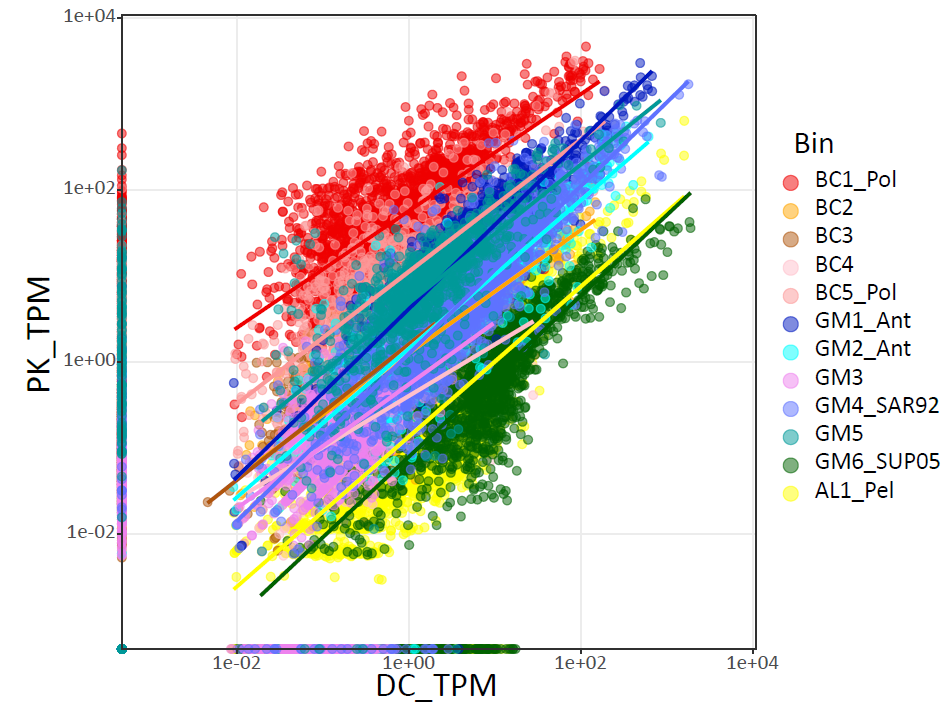


Supplementary Fig. S5. Correlation of gene expression between PK and DC (PK-mRNA-TPM and DC-mRNA-TPM) for selected bins.

Supplementary Fig. S6. COG-based transcriptome analysis of 12 genomes using a scatter plot of mRNA-TPM. Each point represents the relative abundance of a transcript assigned to a COG category. (A) total COGs, (B) transporter-related COGs, (C) GH-related COGs, (D) DMSP-, GB-, and glyoxylate shunt-related COGs, and E) proteorhodopsin-related COGs.

Supplementary Fig. S7. Gene expression levels of representative glycoside hydrolase genes from GM4_SAR92. GH3, glycoside hydrolase 3; GH16, glycoside hydrolase 16; GH42, glycoside hydrolase 42.

Supplementary Fig. S8. Metabolism of dimethylsulfoniopropionate (DMSP), gene expression levels, and gene clusters across the reconstructed genomes. a) relative abundance of dmdA transcripts from each bin, b) organization of the clusters of genes involved in DMSP utilization and c) Pathway for the utilization of DMSP.

1, DMSP demethylase (dmdA); 2, O-acetylhomoserine aminocarboxypropyltransferase; 3, alpha/beta hydrolase fold protein; 4; 3-hydroxyacyl-CoA dehydrogenase (dmdC); 5, acyl-CoA synthetase (dmdB); MMPA, methylmercaptopropionate; MMPA-CoA, 3-methylmercaptopropionyl-CoA, MTA-CoA, methylthioacryloyl-CoA.

Supplementary Figure S9. Synteny of GM1_Ant, GM2_Ant and fosmic clone Ant4D3. Colors are assigned based on the COG classification.

**Reference**

1. Kim JG, Park SJ, Quan ZX, Jung MY, Cha IT, Kim SJ, Kim KH, Yang EJ, Kim YN, Lee SH *et al*: **Unveiling abundance and distribution of planktonic *Bacteria* and *Archaea* in a polynya in Amundsen Sea, Antarctica**. *Environ Microbiol* 2014, **16**(6):1566-1578.
